# Global Prevalence of Antifungal-Resistant *Candida parapsilosis*: A Systematic Review and Meta-Analysis

**DOI:** 10.3390/tropicalmed7080188

**Published:** 2022-08-16

**Authors:** Dina Yamin, Mutiat Hammed Akanmu, Abbas Al Mutair, Saad Alhumaid, Ali A. Rabaan, Khalid Hajissa

**Affiliations:** 1 Department of Medical Microbiology & Parasitology, School of Medical Sciences, Universiti Sains Malaysia, George Town 16150, Malaysia; 2 Department of Biomedicine, School of Health Sciences, Universiti Sains Malaysia, George Town 16150, Malaysia; 3 Research Center, Almoosa Specialist Hospital, Al-Ahsa 36342, Saudi Arabia; 4 College of Nursing, Princess Norah Bint Abdulrahman University, Riyadh 11564, Saudi Arabia; 5 School of Nursing, Wollongong University, Wollongong, NSW 2522, Australia; 6 Nursing Department, Prince Sultan Military College of Health Sciences, Dhahran 33048, Saudi Arabia; 7 Administration of Pharmaceutical Care, Al-Ahsa Health Cluster, Ministry of Health, Al-Ahsa 31982, Saudi Arabia; 8 Molecular Diagnostic Laboratory, Johns Hopkins Aramco Healthcare, Dhahran 31311, Saudi Arabia; 9 College of Medicine, Alfaisal University, Riyadh 11533, Saudi Arabia; 10 Department of Public Health and Nutrition, The University of Haripur, Haripur 22610, Pakistan; 11 Department of Zoology, Faculty of Science and Technology, Omdurman Islamic University, Omdurman P.O. Box 382, Sudan

**Keywords:** *Candida parapsilosis*, prevalence, antifungal drug resistance, global, systematic review, meta-analysis

## Abstract

A reliable estimate of *Candida parapsilosis* antifungal susceptibility in candidemia patients is increasingly important to track the spread of *C. parapsilosis* bloodstream infections and define the true burden of the ongoing antifungal resistance. A systematic review and meta-analysis (SRMA) were conducted aiming to estimate the global prevalence and identify patterns of antifungal resistance. A systematic literature search of the PubMed, Scopus, ScienceDirect and Google Scholar electronic databases was conducted on published studies that employed antifungal susceptibility testing (AFST) on clinical *C. parapsilosis* isolates globally. Seventy-nine eligible studies were included. Using meta-analysis of proportions, the overall pooled prevalence of three most important antifungal drugs; Fluconazole, Amphotericin B and Voriconazole resistant *C. parapsilosis* were calculated as 15.2% (95% CI: 9.2–21.2), 1.3% (95% CI: 0.0–2.9) and 4.7% (95% CI: 2.2–7.3), respectively. Based on study enrolment time, country/continent and AFST method, subgroup analyses were conducted for the three studied antifungals to determine sources of heterogeneity. Timeline and regional differences in *C. parapsilosis* prevalence of antifungal resistance were identified with the same patterns among the three antifungal drugs. These findings highlight the need to conduct further studies to assess and monitor the growing burden of antifungal resistance, to revise treatment guidelines and to implement regional surveillance to prevent further increase in *C. parapsilosis* drug resistance emerging recently.

## 1. Introduction

*Candida* species, the causative agents of the majority of human fungal infections, are becoming a major public health concern [1,2]. In intensive care units (ICUs) around the world, the majority of fungus-related systemic bloodstream infections are caused by species of *Candida*, leading to high death rates and significant healthcare expenses for both governments and hospitalized patients [3,4]. Although *Candida albicans* is the most common and invasive species, its dominance has declined over the last two decades as the number of invasive infections caused by non-albicans *Candida* species has increased [5]. Of these, the *Candida parapsilosis* (*C. parapsilosis*) complex, which consists of the three cryptic species: *C. parapsilosis* sensu stricto, *C. metapsilosis* and *C. orthopsilosis*, is of particular importance, whereas *C. parapsilosis* has the highest prevalence among the cryptic species [6].

Despite the availability of antifungal drugs for treating *Candida* infections, the mortality rate continues to increase [7]. Using a new class of antifungal drugs for infected patients has not improved their prognosis [8]. Drugs such as azoles are used for treating *Candida* infection and have seen an increase in *Candida* resistance due to general and long-term use [9,10]. Indeed, an increase in the rate of azole-resistant *C. parapsilosis* isolates is concerning and requires better understanding of how antifungal drug resistance emerges.

Despite the clinical and economic implications of yeast infection drug resistance, it is still poorly studied in comparison to antibiotic resistance in bacteria pathogens [11]. Although fungal pathogens account for a substantial proportion of bloodstream infection etiologies, they have received relatively less epidemiological attention. Therefore, it is of great significance to conduct a systematic review to understand the global burden of *C. parapsilosis* drug resistant isolates. Accordingly, the aim of this systematic review and meta-analysis (SRMA) is to survey the available data on the antifungal resistance in human’s bloodstream infections caused by *C. parapsilosis*. This will be carried out by systematically retrieving and reviewing this data and generating an updated and comprehensive assessment of the burden of *C. parapsilosis* drug resistance.

## 2. Materials and Methods

### 2.1. Protocol and Reporting Guideline

A precise protocol was agreed upon before the search began, outlining the databases to be searched, eligibility criteria, and all other methodological details. The study was carried out in accordance with the updated guidelines of the Preferred Reporting Items for Systematic Reviews and Meta-analyses (PRISMA) [12] (Table S1).

### 2.2. Search Strategy

To identify studies on the prevalence and pattern of antifungal resistance of *C. parapsilosis* bloodstream infections worldwide, a systematic literature search was conducted in PubMed, Scopus, ScienceDirect and Google Scholar databases. Only articles written in English were included. There were no constraints on study period, study design, or place of publication (Table S2).

### 2.3. Data Management and Study Selection

Initially, all the records identified based on a systematic literature search were exported to Endnote X8 (Clarivate Analytics, London, UK) to be managed. After that, duplicate potential articles were removed by automatic strategy as well as manual search before the screening and assessment of the remaining articles based on title and abstract was independently carried out by two reviewers (D.Y., M.H.A.). Thereafter, the full texts of potential records were downloaded and assessed for eligibility according to the inclusion and exclusion search criteria, by two authors (D.Y., K.H.). Any disagreement or uncertainty were revealed by discussion and consensus.

### 2.4. Data Extraction

The relevant data were extracted from eligible studies by two authors (D.Y. and M.H.A.). Precautions were taken to minimize errors and ensure consistency in data extraction. The following data were extracted to a predesigned Excel spreadsheet: author name, year of publication, study period, study design, country, target group, gender, method of species detection, method of antifungal susceptibility testing (AFST), sample size, total number of cases tested and number of *C. parapsilosis* species resistant cases for several antifungals. Overall, the data from the studies recruited from various geographical locations across the world were analysed.

### 2.5. Quality Assessment

Risk of bias of all selected studies was independently assessed by two authors (D.Y., A.A.M.) using the Joanna Briggs Institute (JBI) critical appraisal checklist for cross-sectional studies [13]. For each article, the final score has been determined as the proportion of ‘yes’ answers for eight items, and subsequently the studies were categorized into “high risk of bias” (low quality) when overall score ≤ 49, “moderate risk of bias” (moderate quality) for score of 50–69% and “low risk of bias” (high quality) if the score ≥ 70%. Disagreements between the reviewers were cleared up by discussion and verification [14,15].

### 2.6. Data Analysis

The data entered in the Excel sheet were analysed using the R package and software. The proportion of resistance to several antifungals was calculated as the number of resistant cases relative to the total number of isolates tested for the relevant antifungal through the use of the Metaprop command. Accordingly, the prevalence of resistance to the studied antifungals (at 95% confidence intervals (CI)) was estimated for each eligible study and subsequently for the world by pooling the antifungal resistance prevalence rates of all included studies using the random-effect model. Heterogeneity between the studies was evaluated by the *I*^2^ statistics in accordance with Cochran’s Q-test. A cut-off value > 75% of *I*^2^ statistic was indication of substantial heterogeneity [16], whilst a *p* value of <0.05 was considered to be a significant degree of heterogeneity. Publication bias was tested graphically using a funnel plot and statistically by Egger’s regression test.

### 2.7. Subgroup and Sensitivity Analysis

For the purpose of exploring the potential sources of heterogeneity, a subgroup analysis was carried out based on different subgroups which are the enrolment time of study, country where the study was conducted, and the AFST method used by using metaprop codes in meta and metafor packages of R (version 3.6.3), in RStudio (version 1.2.5033). Data analysis and the creation of the Forest and Funnel plots were performed.

## 3. Results

### 3.1. Study Selection

**Figure 1:**
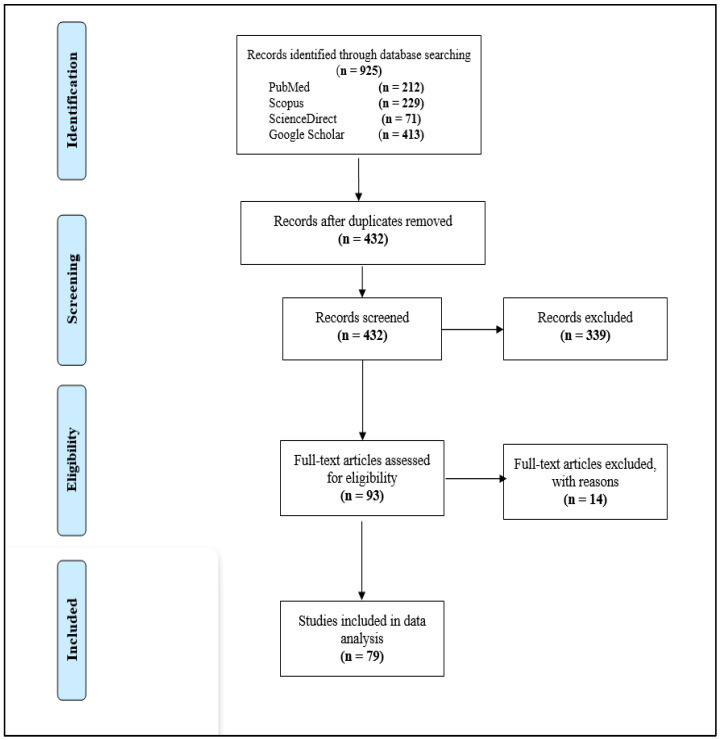
The PRISMA diagram showing the study selection process.

In a flow diagram, Figure 1 shows the results of the literature search and article selection processes. A total of 925 records were initially identified through electronic database searches. After excluding 493 duplicate records, the title and/or abstract of the remaining 432 studies were assessed for inclusion, from which 93 were eligible for full-text screening. Finally, a total of 79 studies met the eligibility criteria and included in this SRMA from which 71 studies were for fluconazole resistance prevalence, 63 for amphotericin B and 58 for voriconazole resistance.

### 3.2. Characteristics of Included Studies

The detailed characteristics of the 79 included studies are summarized in Table 1. Seventy-nine studies published between 1995 and 2022 met the inclusion criteria for antifungal resistance. A total of 14,371 *C. parapsilosis* isolates were identified and subjected to AFST. Fifty (63.3%) of the studies were conducted in America and Asia (24, 26 respectively), 19 (24.1%) in Europe, and 6 (7.6%) in Africa. With respect to the study design, the majority (68.4%, n = 54) were cross-sectional studies, (2.5%, n = 2) prospective or retrospective cohort, (1.3%, n = 1) case control, and the remaining 5 (6.3%) were population- based surveillance studies. Of the 79 articles, 71 provided data on fluconazole resistance, 63 for amphotericin B, 58 for voriconazole, 46 for caspofungin, 40 for itraconazole, 34 for micafungin and anidulafungin each and 23 for Posaconazole. Meta-analysis was performed for the three most important antifungal drugs.

### 3.3. Prevalence of Fluconazole-Resistant C. parapsilosis Isolates

**Figure 2:**
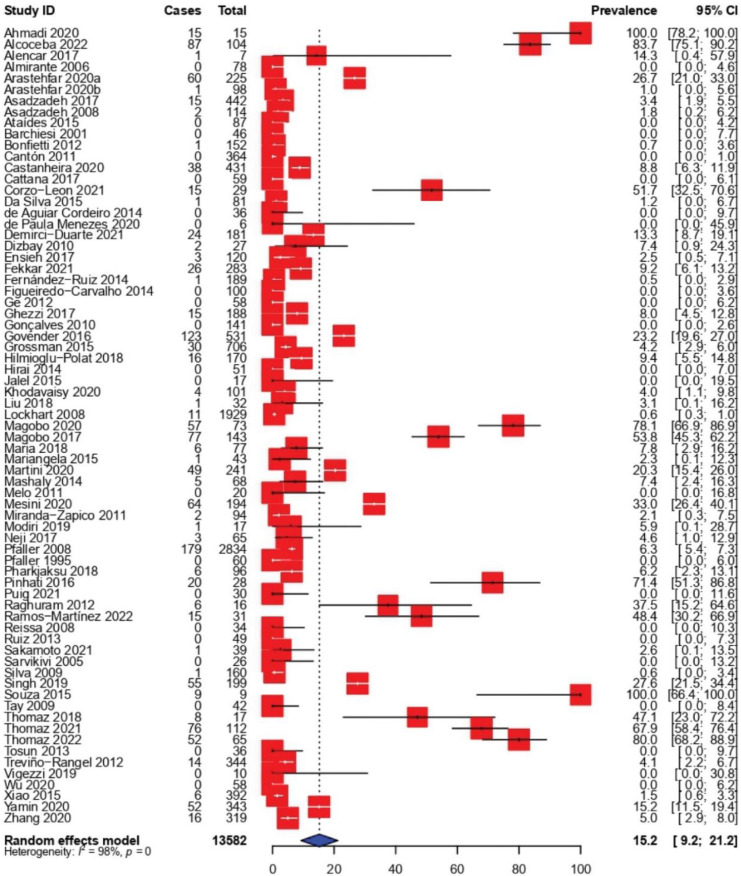
Forest plot representing the pooled prevalence of Fluconazole-Resistant *Candida parapsilosis* isolates.

The pooled prevalence of fluconazole-resistant *C. parapsilosis*, as well as the results of subgroup analysis, are shown in Table 2. The results of the seventy-one studies included in this part of the SRMA show a varied picture of fluconazole resistance rates, ranging from 0% to 100%. In 22 (31.0%) studies, all the identified isolates were susceptible to fluconazole with resistance rates of 0%, while in two other studies, fluconazole resistance was found in 100% of the tested *C. parapsilosis* isolates. The pooled resistance rate of *C. parapsilosis* to fluconazole across the 71 observational studies was estimated to be 15.2% (95% CI: 9.2–21.2) (Figure 2) Significant heterogeneity was observed across all the included studies (*I*^2^ = 98%, *p* < 0.0001). In addition, subgroup analysis was carried out based on enrolment time, country, continent and AFST method to further investigate the potential sources of heterogeneity.

The fluconazole resistance rate has risen dramatically in the last six years, from 11.6% before 2016 to 36.7% in the period from 2016 to 2022. According to the meta-analysis, Africa had the highest prevalence of fluconazole resistance at 27.7% (95% CI: 2.7–52.8), followed by America at 21.2% (95% CI: 7.6–34.7) and Europe at 13.3% (95% CI: 1.3–25.3), while Asia had the lowest frequency of fluconazole resistance at 6.0% (95% CI: 2.9–9.1). Based on the country level (Table S3), the highest prevalence rate of fluconazole-resistant *C. parapsilosis* isolates was reported in South Africa at 51.5%, followed by Mexico at 27.0%, then Brazil at 25.3%. The lowest RA prevalence was reported in Finland and Argentina at 0.0%, followed by Japan and Portugal (0.6%), then China (1.7%). Notably, remarkable differences in fluconazole resistance rate obtained with AFST methods were observed. A slightly high overall estimate was observed when broth microdilution (16.5%; CI: 8.5–24.5) or E-test and broth microdilution (13.0%; 95% CI: 0.5–25.6) were used, while a very low number of *C. parapsilosis* isolates were found to be fluconazole-resistant through DP-Eiken test (0.6%; 95% CI: 0.0–2.9) and all isolates were fluconazole susceptible when MALDI-TOF was used (0.0%; 95% CI: 0.0–11.6).

### 3.4. Prevalence of Amphotericin B-Resistant C. parapsilosis Isolates

**Figure 3:**
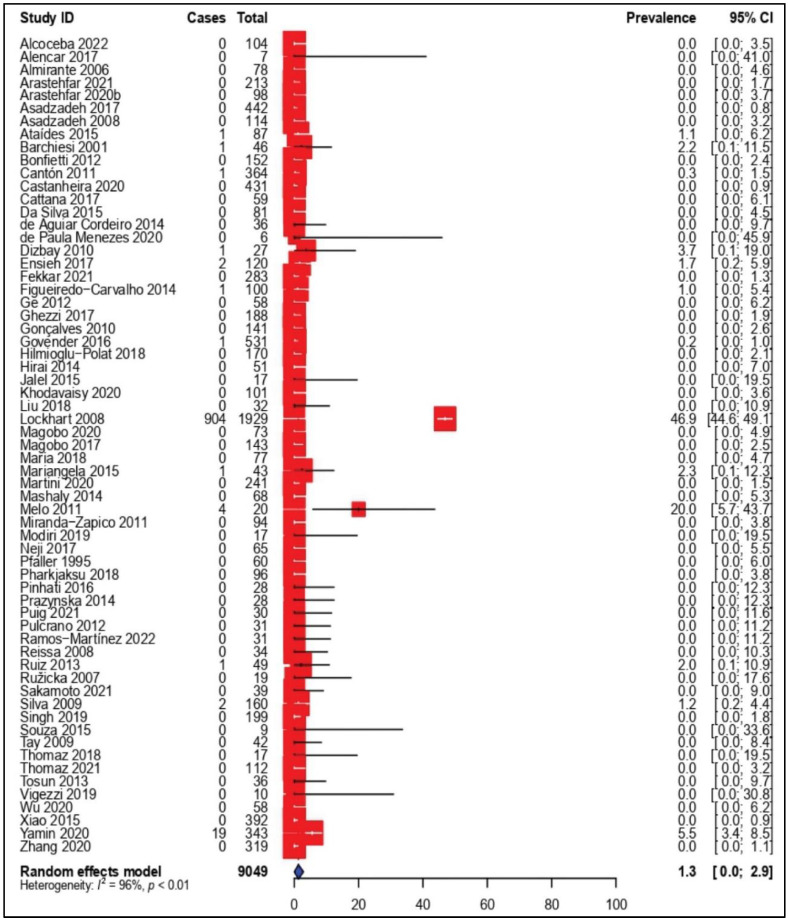
Forest plot representing the pooled prevalence of Amphotericin B-Resistant *Candida parapsilosis* isolates.

The pooled prevalence of amphotericin B-resistant *C. parapsilosis*, as well as the results of subgroup analysis, are shown in Table 2. The results of the 63 studies included in this part of the SRMA show a slightly varied picture of amphotericin B resistance rates, ranging from 0% to 46.9%. In 51 (81.0%) studies, all the identified isolates were susceptible to amphotericin B with resistance rates of 0%, while one study showed the highest amphotericin B resistance rate of 46.9% of the tested *C. parapsilosis* isolates. The pooled resistance rate of *C. parapsilosis* to amphotericin B across the 63 observational studies was estimated to be 1.3% (95% CI: 0.0–2.9) (Figure 3). Significant heterogeneity was observed across all the included studies (*I*^2^ = 96%, *p* < 0.01). Accordingly, subgroup analysis was carried out based on enrolment time, country, continent and AFST method to further investigate the potential sources of heterogeneity.

An amphotericin B resistance rate of 1.6% has been reported before 2016, while it decreased to 0.0% during 2016–2022. According to the meta-analysis, the four continents showed almost the same resistance rate from 0.0–0.2% (95% CI: 0.0–0.7). Based on the country level (Table S3), the highest prevalence rate of amphotericin B-resistant *C. parapsilosis* isolates was reported in Malaysia at 2.9% (95% CI: 0.0–8.3), followed by Portugal at 1.2% (95% CI: 0.2–4.4). Notably, remarkable differences in amphotericin B resistance rate obtained with AFST methods were observed. A slightly high overall estimate was observed when broth microdilution and E-test (5.3%; 95% CI: 0.0–15.5) or E-test (5.3%; 95% CI: 0.0–1.1) were used.

### 3.5. Prevalence of Voriconazole-Resistant C. parapsilosis Isolates

**Figure 4:**
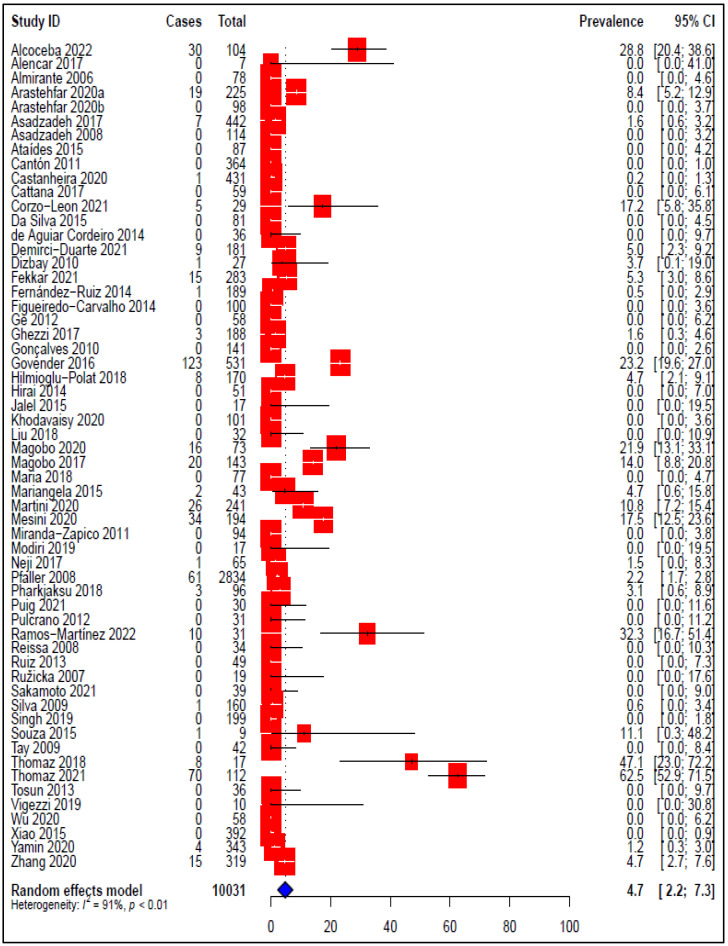
Forest plot representing the pooled prevalence of Voriconazole-Resistant *Candida parapsilosis* isolates.

The pooled prevalence of voriconazole-resistant *C. parapsilosis*, as well as the results of subgroup analysis, are shown in Table 2. The results of the 58 studies included in this section of the SRMA reveal a varied picture of voriconazole resistance rates, ranging from 0.0 to 62.5%. In thirty-one (53.4%) studies, all the identified isolates were susceptible to voriconazole with resistance rates of 0%, while the highest resistance rate was 62.5% of the tested *C. parapsilosis* isolates. The pooled resistance rate of *C. parapsilosis* to voriconazole across the 58 observational studies was estimated to be 4.7% (95% CI: 2.2–7.3) (Figure 4). Significant heterogeneity was observed across all the included studies (*I*^2^ = 91%, *p* < 0.01). Accordingly, subgroup analysis was carried out based on enrolment time, country, continent and AFST method for further investigation of the potential sources of heterogeneity.

The voriconazole resistance rate has increased obviously in the last six years, from 3.2% before 2016 to 17.9% (2016–2022). According to the meta-analysis, Africa had the highest prevalence of voriconazole resistance at 12.0% (95% CI: 2.4–21.6), while Asia had the lowest frequency of voriconazole resistance at 1.2% (95% CI: 0.3–2.0). Based on the country level (Table S3), the highest prevalence rate of voriconazole-resistant *C. parapsilosis* isolates was reported in South Africa at 19.7% (95% CI: 13.5–25.8), followed by Mexico at 17.2% (95% CI: 5.8–35.8), then Brazil at 11.7% (95% CI: 0.0–25.5). The lowest RA prevalence was reported in Argentina, Czechia, India, Iran and Japan at 0.0%. Cleary, remarkable variations in voriconazole resistance rate obtained with AFST methods were noticed. A slightly high overall estimate was observed with E-test and broth microdilution (9.2%; 95% CI: 0.0–22.1), followed by broth microdilution (4.4%; 95% CI: 2.1–6.8).

### 3.6. Quality Assessment and Publication Bias

**Figure 5:**
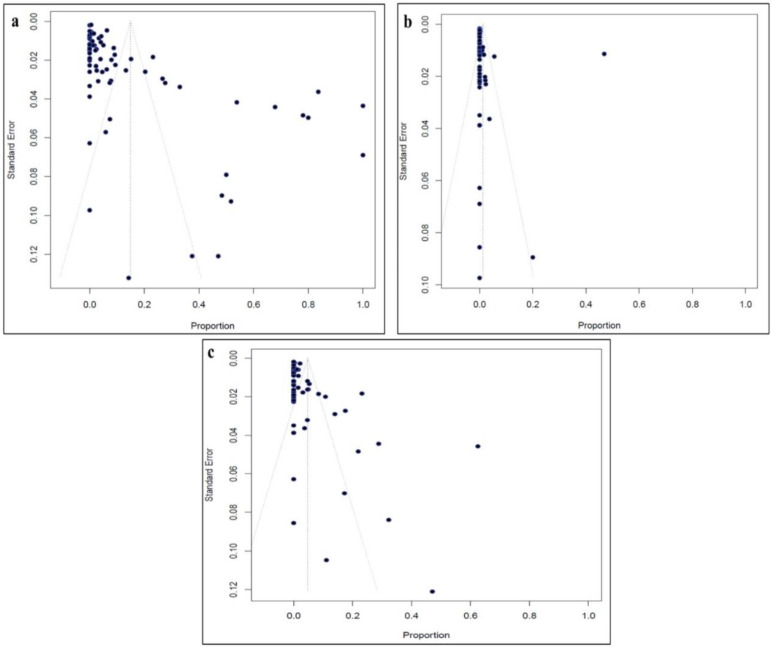
Funnel plots analyzing publication bias among studies evaluated. (**a**) Fluconazole Resistance, (**b**) Amphotericin B Resistance and (**c**) Voriconazole Resistance.

Supplementary Table S4 presents the results of the JBI critical appraisal checklist’s assessment of the 79 included studies’ quality. In summary, 72 (91.1%) of the studies were found to have a low risk of bias, whilst seven (8.9%) were found to have moderate risk of bias. Visual assessment of the symmetrical and asymmetrical funnel plots (Figure 5) revealed the absence and presence of publication bias, respectively. This was statistically confirmed by the Egger’s test for fluconazole, amphotericin B and voriconazole (*p* < 0.0001, 0.1828 and <0.0001 respectively).

## 4. Discussion

Invasive fungal infections caused by nosocomial pathogens such as non-albicans *Candida* including *C. parapsilosis* have emerged, besides a gradual increase in bloodstream infections in healthcare settings, as a result of the widespread administration of broad-spectrum antibiotics, immunosuppressive drugs, and chemotherapy, increased organ transplantation, application of medical support technology, the extension of human life, along with the increase in the prevalence of acquired immune deficiency syndrome (AIDS) [96,97,98]. Antifungal drugs are currently the most effective treatment for *Candida* infections [99,100]. Amphotericin B is considered as a representative of polyene antifungal drugs and has been widely used in the treatment of severe fungal infections [101]. It has been reported that amphotericin B is effective in treating more than 70% of fungal infections. However, it has several clear side effects, mainly nephrotoxicity. The first-generation azoles such as fluconazole and itraconazole show relatively good efficacy [102]. However, the bioavailability of itraconazole differs greatly, and fluconazole resistance develops readily [103]. In contrast, the new triazoles such as voriconazole and Posaconazole show a broader antifungal spectrum, higher bioavailability, and significantly fewer adverse effects than the first-generation triazole drugs. Echinocandins such as caspofungin, micafungin and anidulafungin, inhibit the synthesis of glucan synthase, and inhibit formation of the cell wall, ultimately resulting in cell death [104]. Caspofungin was the first echinocandin to be approved by the US Food and Drug Administration (FDA) and proven to be safe and efficacious against *Candida* species comparatively [105].

Although many authors have broadly addressed the burden of *C. parapsilosis* candidemia and other invasive candidiasis prevalence and antifungal susceptibility profiles, no SRMA summarizes this issue up to date. Here, we conducted a SRMA to address the prevalence of drug-resistant *C. parapsilosis* globally by synthesizing data published to date on *C. parapsilosis* antifungal susceptibility worldwide and provide a point of reference for subsequent studies. The findings of this SRMA were generated by pooling eligible data on the prevalence of antifungal resistant *C. parapsilosis* reported in 79 published studies.

The increasing number of nosocomial *C. parapsilosis* complex infections has raised concerns about conducting antifungal susceptibility tests to optimize clinical treatments. According to CLSI and IDSA, as the first-line drugs, the standardized regimen for *C. parapsilosis* infections treatment are azoles (fluconazole and voriconazole), amphotericin B, then caspofungin. In the present SRMA, data concerning prevalence of fluconazole, amphotericin B and voriconazole resistance are available and sub-grouped based on the enrolment time, country/continent and AFST method.

A total of 71 studies were included, from which the pooled estimate revealed that 15.2% (95% CI 9.2–21.2) of all *C. parapsilosis* cases, 11.6% of cases before 2016 and 36.7% of the cases from 2016 to 2022 had resistance to fluconazole. In the 71 included studies, *C. parapsilosis* clinical isolates were identified using conventional and/or molecular methods. Conventional methods, such as morphological characterization on CHROMagar and Cornmeal agar, and biochemical assimilation on API 20C, ID 32C, Vitek 2 and AUXACOLOR, were the most frequently employed methods, while ITS, D1/D2, PCR-RFLP-SADH, AFLP and MALDI-TOF-MS are among the molecular identification techniques. These studies were conducted in 20 different countries from four continents (Europe, America, Asia and Africa). Based on the available literature, Argentina (0.0%; 95% CI 0.0–2.3) and Finland (0.0%; 95% CI 0.0–13.2) have the lowest prevalence. On the other hand, South Africa (51.5%; 95% CI 20.2–82.7) has the highest prevalence. Variation could be seen between and cross continents. For instance, although South Africa has the highest prevalence, the prevalence of fluconazole resistant *C. parapsilosis* in different counties in the same continent, e.g., Tunisia (3.2%; 95% CI 0.0–7.4) and Egypt (7.4%; 95% CI 2.4–16.3), are dramatically low. It is unclear whether this difference in relative prevalence is the result of different sample size and different geographical regions or both. Data on AFST method of fluconazole resistant *C. parapsilosis* were available. Broth microdilution (16.5%; 95% CI 8.5–24.5) was the highest resistance to fluconazole.

In this study, we also investigated the prevalence of amphotericin B resistant *C. parapsilosis* from a total of 63 studies, from which the pooled estimate showed that 1.3% (95% CI 0.0–2.9), 1.6% of cases before 2016 had been resistance to amphotericin B. The range of prevalence of amphotericin B resistance among the 20 different countries was 0.0–2.9%. Malaysia has the highest prevalence of amphotericin B resistance (2.9%; 95% CI 0.0–8.3). Data on AFST method of amphotericin B resistance showed that the studies using both broth microdilution and E-test have the highest prevalence of amphotericin B resistant *C. parapsilosis* (5.3%; 95% CI 0.0–15.5).

In addition, voriconazole resistance prevalence was determined among 58 studies, in which 4.7% (95% CI 2.2–7.3) of all *C. parapsilosis* cases, 3.2% of cases before 2016 and 17.9% of cases from 2016 to 2022 have resistance to voriconazole. The highest voriconazole resistant *C. parapsilosis* prevalence was reported in Africa (12.0%; 95% CI 2.4–21.6), while the lowest was in Asia (1.2%; 95% CI 0.0–19.2). In Brazil, the prevalence of voriconazole resistance was 11.7% (95% CI 0.0–25.5; *I*^2^ > 75%; *p* value < 0.05), while in South Africa the prevalence was the highest (19.7%; 95% CI 13.5–25.8; *I*^2^ < 75%; *p* value < 0.05). In contrast, the lowest voriconazole resistance prevalence was in China (0.9%; 95% CI 0.0–2.8). The highest prevalence of voriconazole resistance was found when testing using both, broth microdilution and E-test (9.2%; 95% CI 0.0–22.1), similar to amphotericin B resistance prevalence.

Before 2016, the prevalence of fluconazole resistance was the highest, followed by voriconazole resistance, while the prevalence of amphotericin B was the lowest. A similar pattern of antifungal resistance prevalence was found in the period from 2016 to 2022. This finding shows a steady increase in the prevalence of fluconazole resistant *C. parapsilosis* in the last seven years compared to studies conducted before 2016. Regardless of the high rate of fluconazole resistance in many parts of the world, fluconazole remains one of the most effective antifungal drugs. However, the high resistance rate in this study should not be neglected because fluconazole-resistant precursors might accumulate in developing country settings.

Overall, the prevalence of fluconazole resistant *C. parapsilosis* was higher than the prevalence of voriconazole resistant *C. parapsilosis* all over the four continents (ranging from 6.0–27.7, 1.2–12.0 respectively). Consequently, it is recommended to change the first-line treatment of *C. parapsilosis* infections from fluconazole towards voriconazole, especially in Africa, which showed sharply increased fluconazole resistance prevalence. Even though the prevalence of amphotericin B resistance is not significantly high all over the world, it is not recommended as first-line treatment for *C. parapsilosis* infections because it has many side-effects, cannot be administered orally, and due to its toxicity. In contrast, the same scenario of antifungal resistance could not be concluded if countries were compared. Hence, it is worthy to monitor the prevalence of antifungal resistance nationally in different countries to determine the most suitable first-line treatment for each country, because the present viewpoint might be changed if more studies were conducted locally.

Although many novel molecular AFST methods have emerged recently, broth microdilution and disc diffusion (E-test) remain the gold standard AFST assays according to CLSI and EUCAST reports, able to determine antifungal resistance with high sensitivity and specificity.

The overall prevalence of fluconazole resistance *C. parapsilosis* identified in the current study was consistent with the finding of SRMA from India (resistance to fluconazole = 17.63%), amphotericin B = 2.15%, voriconazole = 6.61% [106].

In general, high rates of resistance to fluconazole are unfortunate realities in the majority of *C. parapsilosis* infections. Such high rates could reflect the frequent, unjustified and inadequate extensive usage in general care while having an unknown impact on antifungal susceptibility.

Finally, a key strength of this SRMA is a comprehensive estimation of global *C. parapsilosis* antifungal-resistance, despite the alarming indicative results at the level of continent, in most of the included studies rates was obtained from a smaller sample size. Therefore, expanded surveillance as well as additional studies with a large and systematic sample collection covering various geographical regions across the world are highly recommended.

However, there are several limitations. First, the included studies did not encompass all the countries of the world, and only a limited number of representative studies in the same country were analysed, so the estimated prevalence might not fully reveal the magnitude of drug-resistant *C. parapsilosis* for each county. Second, substantial heterogeneity was observed in the included studies, although this observation is common in meta-analyses estimating prevalence. Finally, the potential effect of gender, age, socioeconomic status, and lifestyle of the included patients on the prevalence of antifungal resistant *C. parapsilosis* could not be analyzed because of the unavailability of data in many of the included studies.

## Figures and Tables

**Table 1:** Detailed characteristics of 79 studies included in SRMA.

No	Study ID [References]	Study Design	Country	No. of Patients		Clinical Isolates Identification Method	AFST Method	Total Isolates Tested	Tested Antifungal
				Male (n)	Female (n)				
1	Ahmadi 2020 [17]	NR	NR	NR	NR	Molecular methods	BMD	15	FLC
2	Alcoceba 2022 [18]	NR	Spain	53	17	Molecular methods	BMD	104	FLC, AMB, POS, VOR, ANF and MCF.
3	Alencar 2017 [19]	Cross sectional	Brazil	NR	NR	Molecular methods	Vitek-2, BMD	7	FLC, AMB, ITC and VOR.
4	Almirante 2006 [20]	Prospective Case Control	Spain	43	35	Conventional methods	BMD	78	FLC, AMB, ITC, VOR and CAS.
5	Arastehfar 2020a [21]	Cross sectional	Turkey	123	91	Both conventional and molecular methods	BMD	225	FLC and VOR.
6	Arastehfar 2021 [22]	Cross sectional	Turkey	NR	NR	Molecular methods	BMD	213	AMB, ANF and MCF.
7	Arastehfar 2020b [23]	Cross sectional	Iran	45	45	Molecular methods	BMD	98	FLC, AMB, ITC, ANF and MCF.
8	Asadzadeh 2017 [24]	Cross sectional	Kuwait	NR	NR	Both conventional and molecular methods	E-test, Vitek-2, BMD	442	FLC, AMB, VOR, CAS and MCF.
9	Asadzadeh 2008 [25]	Cross sectional	Kuwait	NR	NR	Both conventional and molecular methods	E-test	114	FLC, AMB, POS and CAS.
10	Ataídes 2015 [26]	Cross sectional	Brazil	NR	NR	Both conventional and molecular methods	E-test	87	FLC, AMB, ITC, POS, VOR and CAS.
11	Barchiesi 2001 [27]	Cross sectional	Italy	NR	NR	Conventional methods	BMD	46	FLC, AMB and ITC.
12	Bonfietti 2012 [28]	Cross sectional	Brazil	NR	NR	Both conventional and molecular methods	BMD	152	FLC, AMB and ITC.
13	Cantón 2011 [29]	Prospective Cohort	Spain	231	169	Both conventional and molecular methods	Sensititre YeastOne BMD	364	FLC, AMB, ITC, POS, VOR, CAS, ANF and MCF.
14	Castanheira 2020 [30]	Cross sectional	25 countries	NR	NR	Both conventional and molecular methods	BMD	431	FLC, AMB, POS, VOR, CAS, ANF and MCF.
15	Cattana 2017 [31]	Cross sectional	Argentina	NR	NR	Both conventional and molecular methods	BMD	59	FLC, AMB, ITC, VOR, CAS and ANF.
16	Corzo-Leon 2021 [32]	Cross sectional	Mexico	45	29	Both conventional and molecular methods	Vitek-2, BMD	29	FLC and VOR.
17	Da Silva 2015 [33]	Cross sectional	Brazil	27	54	Both conventional and molecular methods	BMD	81	FLC, AMB, ITC and VOR.
18	Davari 2020 [34]	Cross sectional	Iran	NR	NR	Both conventional and molecular methods	BMD	105	CAS, ANF and MCF.
19	de Aguiar Cordeiro 2014 [35]	NR	Italy	NR	NR	Both conventional and molecular methods	BMD	36	FLC, AMB, VOR and CAS.
20	de Paula Menezes 2020 [36]	Cross sectional	Brazil	NR	NR	Both conventional and molecular methods	BMD	6	FLC, AMB and MCF.
21	Demirci-Duarte 2021 [37]	Cross sectional	Turkey	NR	NR	Both conventional and molecular methods	BMD	181	FLC, POS and VOR.
22	Dizbay 2010 [38]	Cross sectional	Turkey	13	14	Conventional methods	BMD	27	FLC, AMB, VOR and CAS.
23	Ensieh 2017 [39]	Cross sectional	Iran	NR	NR	NR	BMD	120	FLC, AMB and ITC.
24	Fekkar 2021 [40]	Cross sectional	France	NR	NR	Molecular methods	E-test, BMD	283	FLC, AMB, ITC, POS, VOR, CAS and MCF.
25	Fernández-Ruiz 2014 [41]	Cross sectional	Spain	127	63	Both conventional and molecular methods	BMD	189	FLC, VOR, ANF and MCF.
26	Figueiredo-Carvalho 2014 [42]	Cross sectional	Brazil	NR	NR	Both conventional and molecular methods	E-test, Vitek-2, BMD	100	FLC, AMB, ITC, VOR and CAS.
27	Garcia-Effron 2012 [43]	Cross sectional	Spain	179	108	Both conventional and molecular methods	BMD	287	CAS, ANF and MCF.
28	Ge 2012 [44]	Cross sectional	China	NR	NR	Both conventional and molecular methods	BMD	58	FLC, AMB, ITC, VOR and MCF.0
29	Ghezzi 2017 [45]	Retrospective cohort	Italy	264	188	Both conventional and molecular methods	BMD	188	FLC, AMB, ITC, POS, VOR, CAS, ANF and MCF.
30	Gonçalves 2010 [46]	Cross sectional	Brazil	86	60	Both conventional and molecular methods	BMD	141	FLC, AMB, ITC, VOR and CAS.
31	Govender 2016 [47]	Cross sectional	South Africa	279/513	234/513	Both conventional and molecular methods	BMD	531	FLC, AMB, ITC, POS, VOR, CAS, ANF and MCF.
32	Grossman 2015 [48]	Cross sectional	USA	NR	NR	Both conventional and molecular methods	E-test, BMD	706	FLC.
33	Hilmioğlu-Polat 2018 [49]	Cross sectional	Turkey	NR	NR	Molecular methods	BMD	170	FLC, AMB, VOR, CAS and ANF.
34	Hirai 2014 [50]	Cross sectional	Japan	37/51	14/51	Conventional methods	DP-Eiken	51	FLC, AMB, ITC, VOR and MCF.
35	Jalel 2015 [51]	Cross sectional	Tunisia	NR	NR	Both conventional and molecular methods	E-test	17	FLC, AMB, ITC and VOR.
36	Khan 2011 [52]	Cross sectional	Kuwait	NR	NR	Both conventional and molecular methods	E-test	86	CAS and ANF.
37	Khodavaisy 2020 [53]	Cross sectional	Iran	34	67	Molecular methods	BMD	101	FLC, AMB, ITC, POS, VOR, CAS, ANF and MCF.
38	Liu 2018 [54]	Cross sectional	China	22	10	NR	E-test	32	FLC, AMB, VOR and CAS.
39	Lockhart 2008 [55]	Cross sectional	25 countries	NR	NR	Both conventional and molecular methods	E-test, BMD	1929	FLC, AMB, CAS, ANF and MCF.
40	Magobo 2020 [56]	Cross sectional	South Africa	NR	NR	Both conventional and molecular methods	NR	73	FLC, AMB, ITC, POS, VOR, CAS, ANF and MCF.
41	Magobo 2017 [57]	Cross sectional	South Africa	NR	NR	Both conventional and molecular methods	E-test, BMD	143	FLC, AMB, ITC, POS, VOR, CAS, ANF and MCF.
42	Maria 2018 [58]	Cross sectional	India	42	35	Both conventional and molecular methods	E-test, Vitek-2, BMD	77	FLC, AMB, VOR, CAS and MCF.
43	Mariangela 2015 [59]	Cross sectional	Brazil	NR	NR	Both conventional and molecular methods	Vitek-2, BMD	43	FLC, AMB, ITC, VOR and CAS.
44	Martini 2020 [60]	Cross sectional	Italy	NR	NR	Molecular methods	Sensititre YeastOne, BMD	241	FLC, AMB, ITC, POS, VOR, CAS, ANF and MCF.
45	Mashaly 2014 [61]	Cross sectional	Egypt	29	39	Both conventional and molecular methods	E-test	68	FLC, AMB and ITC.
46	Melo 2011 [62]	NR	Brazil	NR	NR	Both conventional and molecular methods	NR	20	FLC and AMB.
47	Mesini 2020 [63]	Cross sectional	Italy	386	274	Both conventional and molecular methods	Sensititre YeastOne, BMD	194	FLC, VOR, CAS, ANF and MCF.
48	Miranda-Zapico 2011 [64]	Cross sectional	Spain	NR	NR	Both conventional and molecular methods	Sensititre YeastOne, BMD	94	FLC, AMB, ITC, POS, VOR, CAS, ANF and MCF.
49	Modiri 2019 [65]	NR	Iran	NR	NR	Molecular methods	BMD	17	FLC, AMB, ITC, POS, VOR and CAS.
50	Neji 2017 [66]	Cross sectional	Tunisia	NR	NR	Both conventional and molecular methods	Sensititre YeastOne, BMD	65	FLC, AMB, ITC, VOR and CAS.
51	Pfaller 2008 [67]	Surveillance	Many countries	NR	NR	NR	E-test, BMD	2834	FLC VOR, CAS, ANF and MCF.
52	Pfaller 1995 [68]	NR	USA	NR	NR	Molecular methods	BMD	60	FLC, AMB and ITC.
53	Pharkjaksu 2018 [69]	Cross sectional	Thailand	NR	NR	Molecular methods	Sensititre YeastOne, BMD	96	FLC, AMB, ITC, POS, VOR, CAS, ANF and MCF.
54	Pinhati 2016 [70]	Cross sectional	Brazil	25	15	Both conventional and molecular methods	Vitek-2, BMD	28	FLC, AMB and ANF.
55	Prażyńska 2014 [71]	Cross sectional	Poland	NR	NR	Conventional methods	BMD	28	AMB.
56	Puig 2021 [72]	Cross sectional	Spain	NR	NR	Both conventional and molecular methods	MALDI-TOF	30	FLC, AMB, ITC, POS, VOR, CAS, ANF and MCF.
57	Pulcrano 2012 [73]	NR	Italy	NR	NR	Both conventional and molecular methods	BMD	31	AMB and VOR.
58	Raghuram 2012 [74]	Cross sectional	USA	NR	NR	NR	NR	16	FLC and CAS.
59	Ramos-Martínez 2022 [75]	Cross sectional	Spain	61	27	Both conventional and molecular methods	BMD	31	FLC, AMB, POS, VOR and CAS.
60	Reissa 2008 [76]	NR	USA	NR	NR	Both conventional and molecular methods	E-test, BMD	34	FLC, AMB, ITC, POS, VOR and CAS.
61	Roberto 2020 [77]	NR	Brazil	NR	NR	Molecular methods	MALDI-TOF-MS, BMD	20	CAS, ANF and MCF.
62	Ruiz 2013 [78]	Cross sectional	Brazil	NR	NR	Molecular methods	E-test	49	FLC, AMB, ITC, VOR and CAS.
63	Růžička 2007 [79]	NR	Czechia	NR	NR	Conventional methods	BMD	19	AMB, ITC and VOR.
64	Sakamoto 2021 [80]	Cross sectional	Japan	96	51	Conventional methods	DP-Eiken	39	FLC, AMB, ITC, VOR, CAS and MCF.
65	Sarvikivi 2005 [81]	Cross sectional	Finland	NR	NR	Both conventional and molecular methods	BMD	26	FLC.
66	Silva 2009 [82]	Cross sectional	Portugal	NA	NA	Both conventional and molecular methods	BMD	160	FLC, AMB, POS, VOR, CAS and ANF.
67	Singh 2019 [83]	Surveillance	India	NR	NR	Both conventional and molecular methods	BMD	199	FLC, AMB, ITC, POS and VOR.
68	Souza 2015 [84]	Surveillance	Brazil	NR	NR	Both conventional and molecular methods	Vitek-2, BMD	9	FLC, AMB, VOR and ANF.
69	Tay 2009 [85]	NR	Malaysia	NR	NR	Both conventional and molecular methods	E-test	42	FLC, AMB, ITC, KET and VOR.
70	Thomaz 2018 [86]	NR	Brazil	NR	NR	Both conventional and molecular methods	BMD	17	FLC, AMB, VOR, ANF and MCF.
71	Thomaz 2021 [87]	NR	Brazil	NR	NR	Molecular methods	E-test, BMD	112	FLC, AMB, VOR, ANF and MCF.
72	Thomaz 2022 [88]	NR	Brazil	NR	NR	Molecular methods	Disk Diffusion	65	FLC.
73	Tosun 2013 [89]	NR	Turkey	NR	NR	Both conventional and molecular methods	BMD	36	FLC, AMB, VOR, CAS and ANF.
74	Treviño-Rangel 2012 [90]	NR	Mexico	NR	NR	Both conventional and molecular methods	BMD	344	FLC CAS, ANF and MCF.
75	Vigezzi 2019 [91]	NR	Argentina	NR	NR	Both conventional and molecular methods	BMD	10	FLC, AMB, ITC, POS, VOR, CAS and ANF.
76	Wu 2020 [92]	Cross sectional	China	33	25	NR	NR	58	FLC, AMB, ITC, VOR, and MCF.
77	Xiao 2015 [93]	Surveillance	China	NR	NR	Conventional methods	E-test, Sensititre YeastOne BMD	392	FLC, AMB, ITC, POS, VOR, CAS, ANF and MCF.
78	Yamin 2020 [94]	Cross sectional	Malaysia	NR	NR	Conventional methods	NA	343	FLC, AMB, VOR, and CAS.
79	Zhang 2020 [95]	Surveillance	China	232	87	Molecular methods	Sensititre YeastOne BMD	319	FLC, AMB, ITC, POS, VOR, CAS, ANF and MCF.

AFST: Antifungal Susceptibility testing, BMD: Broth Microdilution, FLC: Fluconazole, AMB: Amphotericin B, ITC: Itraconazole, KET: Ketoconazole, POS: Posaconazole, VOR: Voriconazole, CAS: Caspofungin, ANF: Anidulafungin, MCF: Micafungin, NR: Not reported.

**Table 2:** Pooled *C. parapsilosis* antifungal resistance in different subgroups.

Subgroups		Prevalence of Antifungal Resistance [95% CIs] (%)	No. of Studies Analysed	Total No. of Subjects	Heterogeneity		Publication Bias, Egger’s Test (*p*-Value)
					*I* ^2^	*p*-Value	
Fluconazole							
Total		15.2 [9.2; 21.2]	71	13,582	98%	<0.0001	<0.0001
Enrolment time	Before 2016	11.6 [4.9; 18.3]	43	10,244	97%	<0.01	0.0002
	2016–2022	36.7 [10.9; 62.6]	8	1126	99%	<0.01	NA
Continent	Europe	13.3 [1.3–25.3]	15	2064	98%	<0.01	0.0439
	America	21.2 [7.6–34.7]	23	1831	97%	<0.01	0.0116
	Asia	6.0 [2.9–9.1]	23	3237	90%	<0.01	0.0116
	Africa	27.7 [2.7–52.8]	6	897	98%	<0.01	NA
AFST method	BrothMicrodilution	16.5 [8.5–24.5]	43	5107	98%	<0.0001	<0.0001
	E-test and Broth Microdilution	13.0 [0.5–25.6]	12	7371	98%	<0.01	0.0315
	E-test	11.3 [0.0–30.2]	8	474	97%	<0.01	NA
	DP-Eiken	0.6 [0.0–2.9]	2	90	0%	0.37	NA
	MALDI-TOF	0.0 [0.0–11.6]	1	30	NA	NA	NA
Amphotericin B							
Total		1.3 [0.0–2.9]	63	9049	96%	<0.01	0.1828
Enrolment time	Before 2016	1.6 [0.0–4.1]	40	6023	98%	<0.0001	0.2710
	2016–2022	0.0 [0.0–0.2]	8	1138	0	1	NA
Continent	Europe	0.1 [0.0–0.4]	15	1733	0	1	0.3617
	America	0.2 [0.0–0.7]	18	1015	0	0.95	0.0419
	Asia	0.0 [0.0–0.2]	22	3044	9%	0.34	0.1135
	Africa	0.2 [0.0–0.05]	6	897	0%	1	NA
AFST method	Broth Microdilution	0.1 [0.0–0.2]	40	4514	0	1	0.0936
	E-test and Broth Microdilution	5.3 [0.0–15.5]	9	3512	100	<0.0001	NA
	E-test	5.3 [0.0–1.1]	7	409	0	0.95	NA
	DP-Eiken	0.0 [0.0–2.1]	2	90	0	1	NA
	MALDI-TOF	0.0 [0.0–11.6]	1	30	NA	Na	NA
Voriconazole							
Total		4.7 [2.2; 7.3]	58	10,031	91%	<0.01	<0.0001
Enrolment time	Before 2016	3.2 [1.2–5.2]	37	8030	93%	<0.01	0.0078
	2016–2022	17.9 [0.2–35.6]	7	1132	98%	<0.01	NA
Continent	Europe	5.3 [0.8–9.7]	15	2042	90%	<0.01	0.0054
	America	9.2 [0.0–19.2]	14	778	94%	<0.01	0.0569
	Asia	1.2 [0.3–2.0]	22	3117	67%	<0.01	0.0120
	Africa	12.0 [2.4–21.6]	5	829	96%	<0.01	NA
AFST method	Broth Microdilution	4.4 [2.1–6.8]	37	4679	90%	<0.01	0.0002
	E-test and Broth Microdilution	9.2 [0.0–22.1]	9	4417	97%	<0.01	NA
	E-test	0.0 [0.0–0.8]	6	341	0%	1	NA
	DP-Eiken	0.0 [0.0–2.1]	2	90	0	1	NA
	MALDI-TOF	0.0 [0.0–11.6]	1	30	NA	NA	Na

AFST: antifungal susceptibility testing; CIs: Confidence intervals; NA: Not applicable.

## Data Availability

The data generated in this study are available within the manuscript and Supplementary Materials.

## Supplementary Materials

The following supporting information can be downloaded at: https://www.mdpi.com/article/10.3390/tropicalmed7080188/s1, Table S1: PRISMA checklist. Table S2: Detailed Search Strategy. Table S3: Pooled *C. parapsilosis* antifungal resistance in different countries. Table S4: Quality assessment of the included studies.

## References

[1] Kotey F.C., Dayie N.T., Tetteh-Uarcoo P.B., Donkor E.S. (2021). *Candida* Bloodstream Infections: Changes in Epidemiology and Increase in Drug Resistance. Infect. Dis. Res. Treat..

[2] De Oliveira Santos G.C., Vasconcelos C.C., Lopes A.J.O., de Sousa Cartágenes M.D.S., Filho A.K.D.B., do Nascimento F.R.F., Ramos R., Pires E.R.R.B., de Andrade M.S., Rocha F.M.G. (2018). *Candida* Infections and Therapeutic Strategies: Mechanisms of Action for Traditional and Alternative Agents. Front. Microbiol..

[3] Kett D.H., Azoulay E., Echeverria P.M., Vincent J.-L. (2011). *Candida* bloodstream infections in intensive care units: Analysis of the extended prevalence of infection in intensive care unit study. Crit. Care Med..

[4] Wan Ismail W.N.A., Jasmi N., Khan T.M., Hong Y.H., Neoh C.F. (2020). The Economic Burden of Candidemia and Invasive Candidiasis: A Systematic Review. Value Health Reg. Issues.

[5] Lamoth F., Lockhart S.R., Berkow E.L., Calandra T. (2018). Changes in the epidemiological landscape of invasive candidiasis. J. Antimicrob. Chemother..

[6] Tavanti A., Davidson A.D., Gow N.A., Maiden M.C., Odds F.C. (2005). *Candida orthopsilosis* and *Candida metapsilosis* spp. nov. to replace *Candida parapsilosis* groups II and III. J. Clin. Microbiol..

[7] Pristov K.E., Ghannoum M.A. (2019). Resistance of *Candida* to azoles and echinocandins worldwide. Clin. Microbiol. Infect..

[8] Armstrong-James D., Brown G.D., Netea M.G., Zelante T., Gresnigt M.S., van de Veerdonk F.L., Levitz S.M. (2017). Immunotherapeutic approaches to treatment of fungal diseases. Lancet Infect. Dis..

[9] Marak M.B., Dhanashree B. (2018). Antifungal susceptibility and biofilm production of *Candida* spp. isolated from clinical samples. Int. J. Microbiol..

[10] Pahwa N., Kumar R., Nirkhiwale S., Bandi A. (2014). Species distribution and drug susceptibility of *Candida* in clinical isolates from a tertiary care centre at Indore. Indian J. Med. Microbiol..

[11] Ksiezopolska E., Gabaldón T.J.G. (2018). Evolutionary emergence of drug resistance in *Candida* opportunistic pathogens. Genes.

[12] Page M.J., Moher D., Bossuyt P.M., Boutron I., Hoffmann T.C., Mulrow C.D., Shamseer L., Tetzlaff J.M., Akl E.A., Brennan S.E. (2021). PRISMA 2020 explanation and elaboration: Updated guidance and exemplars for reporting systematic reviews. BMJ.

[13] Munn Z., Moola S., Lisy K., Riitano D., Tufanaru C. (2015). Methodological guidance for systematic reviews of observational epidemiological studies reporting prevalence and cumulative incidence data. Int. J. Evid.-Based Healthc..

[14] Hajissa K., Marzan M., Idriss M., Islam A. (2021). Prevalence of Drug-Resistant Tuberculosis in Sudan: A Systematic Review and Meta-Analysis. Antibiotics.

[15] Hajissa K., Islam M.A., Hassan S.A., Zaidah A.R., Ismail N., Mohamed Z. (2022). Seroprevalence of SARS-CoV-2 Antibodies in Africa: A Systematic Review and Meta-Analysis. Int. J. Environ. Res. Public Health.

[16] Hajissa K., Islam A., Sanyang A.M., Mohamed Z. (2022). Prevalence of intestinal protozoan parasites among school children in Africa: A systematic review and meta-analysis. PLoS Negl. Trop. Dis..

[17] Ahmadi A., Mahmoudi S., Rezaie S., Hashemi S.J., Dannaoui E., Badali H., Ghaffari M., Aala F., Izadi A., Maleki A. (2020). In vitro synergy of echinocandins with triazoles against fluconazole-resistant *Candida parapsilosis* complex isolates. J. Glob. Antimicrob. Resist..

[18] Alcoceba E., Gómez A., Lara-Esbrí P., Oliver A., Beltrán A.F., Ayestarán I., Muñoz P., Escribano P., Guinea J. (2022). Fluconazole-resistant *Candida parapsilosis* clonally related genotypes: First report proving the presence of endemic isolates harbouring the Y132F *ERG11* gene substitution in Spain. Clin. Microbiol. Infect..

[19] De Alencar D.D.S.O., de Sousa Tsujisaki R.A., Spositto F.L.E., de Oliveira Nunes M., de Almeida A.A., dos Anjos Martins M., de Souza CarvalhoMelhem M., Chang M.R. (2017). Candidaemia due to *Candida parapsilosis* species complex at a hospital in Brazil: Clinical characteristics and antifungal susceptibility profile. Rev. Iberoam. Micol..

[20] Almirante B., Rodríguez D., Cuenca-Estrella M., Almela M., Sanchez F., Ayats J., Alonso-Tarres C., Rodriguez-Tudela J.L., Pahissa A., Barcelona Candidemia Project Study Group (2006). Epidemiology Risk Factors, and Prognosis of *Candida parapsilosis* Bloodstream Infections: Case-Control Population-Based Surveillance Study of Patients in Barcelona, Spain, from 2002 to 2003. J. Clin. Microbiol..

[21] Arastehfar A., Daneshnia F., Hilmioğlu-Polat S., Fang W., Yaşar M., Polat F., Metin D.Y., Rigole P., Coenye T., Ilkit M. (2020). First report of candidemia clonal outbreak caused by emerging fluconazole-resistant *Candida parapsilosis* isolates harboring Y132F and/or Y132F + K143R in Turkey. Antimicrob. Agents Chemother..

[22] Arastehfar A., Daneshnia F., Hilmioglu-Polat S., Ilkit M., Yasar M., Polat F., Metin D.Y., Dokumcu Ü.Z., Pan W., Hagen F. (2021). Genetically related micafungin-resistant *Candida parapsilosis* blood isolates harbouring novel mutation R658G in hotspot 1 of Fks1p: A new challenge?. J. Antimicrob. Chemother..

[23] Arastehfar A., Daneshnia F., Najafzadeh M.J., Hagen F., Mahmoudi S., Salehi M., Zarrinfar H., Namvar Z., Zareshahrabadi Z., Khodavaisy S. (2020). Evaluation of molecular epidemiology, clinical characteristics, antifungal susceptibility profiles, and molecular mechanisms of antifungal resistance of Iranian *Candida parapsilosis* species complex blood isolates. Front. Cell. Infect. Microbiol..

[24] Asadzadeh M., Ahmad S., Al-Sweih N., Khan Z. (2017). Epidemiology and molecular basis of resistance to fluconazole among clinical *Candida parapsilosis* isolates in Kuwait. Microb. Drug Resist..

[25] Asadzadeh M., Al-Sweih N.A., Ahmad S., Khan Z.U. (2008). Antifungal susceptibility of clinical *Candida parapsilosis* isolates in Kuwait. Mycoses.

[26] Ataides F.S., Costa C.R., Souza L.K.H., Fernandes O.D.L., Jesuino R.S.A., Silva M.D.R.R. (2015). Molecular identifi cation and antifungal susceptibility profi les of *Candida parapsilosis* complex species isolated from culture collection of clinical samples. Rev. Soc. Bras. Med. Trop..

[27] Barchiesi F., Di Francesco L.F., Arzeni D., Caselli F., Simonetti O., Cellini A., Giacometti A., Offidani A., Scalise G. (2001). Electrophoretic karyotyping and antifungal susceptibility patterns of *Candida parapsilosis* clinical isolates causing deep and superficial fungal infections. Mycopathologia.

[28] Bonfietti L.X., Martins M.D.A., Szeszs M.W., Pukiskas S.B.S., Purisco S.U., Pimentel F.C., Pereira G.H., Silva D.C., Oliveira L., Melhem M.D.S.C. (2012). Prevalence, distribution and antifungal susceptibility profiles of *Candida parapsilosis*, *Candida orthopsilosis* and *Candida metapsilosis* bloodstream isolates. J. Med. Microbiol..

[29] Cantón E., Pemán J., Quindós G., Eraso E., Miranda-Zapico I., Álvarez M., Merino P., Campos-Herrero I., Marco F., de la Pedrosa E.G.G. (2011). Prospective multicenter study of the epidemiology, molecular identification, and antifungal susceptibility of *Candida parapsilosis*, *Candida orthopsilosis*, and *Candida metapsilosis* isolated from patients with candidemia. Antimicrob. Agents Chemother..

[30] Castanheira M., Deshpande L.M., Messer S.A., Rhomberg P.R., Pfaller M.A. (2020). Analysis of global antifungal surveillance results reveals predominance of Erg11 Y132F alteration among azole-resistant *Candida parapsilosis* and *Candida tropicalis* and country-specific isolate dissemination. Int. J. Antimicrob. Agents.

[31] Cattana M.E., Dudiuk C., Fernández M., Rojas F., Alegre L., Córdoba S., Garcia-Effron G., Giusiano G. (2017). Identification of Candida parapsilosis sensu lato in pediatric patients and antifungal susceptibility testing. J. Antimicrob. Chemother..

[32] Corzo-Leon D.E., Peacock M., Rodriguez-Zulueta P., Salazar-Tamayo G.J., MacCallum D.M. (2021). General hospital outbreak of invasive candidiasis due to azole-resistant Candida parapsilosis associated with an Erg11 Y132F mutation. Med. Mycol..

[33] Da Silva B.V., Silva L.B., de Oliveira D.B.C., da Silva P.R., Ferreira-Paim K., Andrade-Silva L.E., Silva-Vergara M.L., Andrade A.A. (2015). Species distribution, virulence factors, and antifungal susceptibility among Candida parapsilosis complex isolates recovered from clinical specimens. Mycopathologia.

[34] Davari A., Haghani I., Hassanmoghadam F., Nabili M., Shokohi T., Hedayati M.T., Shabanzadeh S., Moazeni M. (2020). Echinocandin resistance in Candida parapsilosis sensu stricto: Role of alterations in CHS3, FKS1 and Rho gene expression. J. Glob. Antimicrob. Res..

[35] de Aguiar Cordeiro R., de Brito Macedo R., Teixeira C.E.C., de Farias Marques F.J., Bandeira T.d.J.P.G., Moreira J.L.B., Brilhante R.S.N., Rocha M.F.G., Sidrim J.J.C. (2014). The calcineurin inhibitor cyclosporin A exhibits synergism with antifungals against Candida parapsilosis species complex. J. Med. Microb..

[36] de Paula Menezes R., de Oliveira Melo S.G., Bessa M.A.S., Silva F.F., Alves P.G.V., Araújo L.B., Penatti M.P.A., Abdallah V.O.S., von Dollinger de Brito Röder D., dos Santos Pedroso R. (2020). Candidemia by Candida parapsilosis in a neonatal intensive care unit: Human and environmental reservoirs, virulence factors, and antifungal susceptibility. Braz. J. Microb..

[37] Demirci-Duarte S., Arikan-Akdagli S., Gülmez D. (2021). Species distribution, azole resistance and related molecular mechanisms in invasive Candida parapsilosis complex isolates: Increase in fluconazole resistance in 21 years. Mycoses.

[38] Dizbay M., Fidan I., Kalkanci A., Sari N., Yalcin B., Kustimur S., Arman D. (2010). High incidence of Candida parapsilosis candidaemia in non-neutropenic critically ill patients: Epidemiology and antifungal susceptibility. Scand. J. Infect. Dis..

[39] Ensieh L., Ali G., Parivash K., Farideh Z., Hossein M., Rasoul M., Fatemeh N., Sassan R. (2017). Regulation of ERG3, ERG6, and ERG11 genes in antifungal-resistant isolates of candida parapsilosis. Iran. Biomed. J..

[40] Fekkar A., Blaize M., Bouglé A., Normand A.-C., Raoelina A., Kornblum D., Kamus L., Piarroux R., Imbert S. (2021). Hospital outbreak of fluconazole-resistant Candida parapsilosis: Arguments for clonal transmission and long-term persistence. Antimicrob. Ag. Chemother..

[41] Fernández-Ruiz M., Aguado J., Almirante B., Lora-Pablos D., Padilla B., Puig-Asensio M., Montejo M., García-Rodríguez J., Pemán J., Ruiz Pérez de Pipaón M. (2014). CANDIPOP Project, GEIH-GEMICOMED (SEIMC), REIPI. 2014. Initial use of echinocandins does not negatively influence outcome in Candida parapsilosis bloodstream infection: A propensity score analysis. Clin. Infect. Dis..

[42] Figueiredo-Carvalho M.H.G., Barbedo L.S., Oliveira M.M., Brito-Santos F., Almeida-Paes R., Zancopé-Oliveira R.M. (2014). Comparison of commercial methods and the CLSI broth microdilution to determine the antifungal susceptibility of Candida parapsilosis complex bloodstream isolates from three health institutions in Rio de Janeiro, Brazil. Mycopathologia.

[43] Garcia-Effron G., Canton E., Pemán J., Dilger A., Romá E., Perlin D.S. (2012). Epidemiology and echinocandin susceptibility of Candida parapsilosis sensu lato species isolated from bloodstream infections at a Spanish university hospital. J. Antimicrob. Chemother..

[44] Ge Y.P., Boekhout T., Zhan P., Lu G.X., Shen Y.N., Li M., Shao H.F., Liu W.D. (2012). Characterization of the Candida parapsilosis complex in East China: Species distribution differs among cities. Med. Mycol..

[45] Ghezzi M.C., Brunetti G., Visconti V., Giordano A., Raponi G. (2017). Candidaemia in a tertiary care academic hospital in Italy. The impact of C. parapsilosis complex on the species distribution and antifungal susceptibility. J. Med. Microb..

[46] Gonçalves S., Amorim C., Nucci M., Padovan A., Briones M.R., Melo A.S., Colombo A.L. (2010). Prevalence rates and antifungal susceptibility profiles of the Candida parapsilosis species complex: Results from a nationwide surveillance of candidaemia in Brazil. Clin. Microb. Infect..

[47] Govender N., Patel J., Magobo R., Naicker S., Wadula J., Whitelaw A., Coovadia Y., Kularatne R., Govind C., Lockhart S. (2016). TRAC-South Africa group. Emergence of azole-resistant Candida parapsilosis causing bloodstream infection: Results from laboratory-based sentinel surveillance in South Africa. J. Antimicrob. Chemother..

[48] Grossman N.T., Pham C.D., Cleveland A.A., Lockhart S.R. (2015). Molecular mechanisms of fluconazole resistance in Candida parapsilosis isolates from a US surveillance system. Antimicrob. Age. Chemother..

[49] Hilmioğlu-Polat S., Sharifynia S., Öz Y., Aslan M., Gündoğdu N., Serin A., Rafati H., Mohammadi F., Yeşim-Metin D., Döğen A. (2018). Genetic diversity and antifungal susceptibility of Candida parapsilosis sensu stricto isolated from bloodstream infections in Turkish patients. Mycopathologia.

[50] Hirai Y., Asahata S., Ainoda Y., Goto A., Fujita T., Totsuka K. (2014). Nosocomial Candida parapsilosis candidaemia: Risk factors, antifungal susceptibility and outcome. J. Hosp. Infect..

[51] Jalel B., Fatma S. (2015). Molecular Identification and Antifungal Susceptibility of Candida parapsilosis sensu stricto, Candida orthopsilosis, and Candida metapsilosis in Sousse Region, Tunisia. Med. Mycol..

[52] Khan Z., Ahmad S., Joseph L., Chandy R., Theyyathel A. (2011). Comparative In Vitro Susceptibility of Clinical Isolates of Candida parapsilosis Complex and Other Candida Species to Caspofungin and Anidulafungin by Etest. J. Chemother..

[53] Khodavaisy S., Badali H., Meis J.F., Modiri M., Mahmoudi S., Abtahi H., Salehi M., Dehghan Manshadi S.A., Aala F., Agha Kuchak Afshari S. (2020). Comparative in vitro activities of seven antifungal drugs against clinical isolates of Candida parapsilosis complex. J. Mycol. Med..

[54] Liu Y., Kang M., Ye H., Zong Z., Lv X. (2018). Analysis on clinical characteristics and drug resistance of Candida parapsilosis bloodstream infections in West China Hospital, China, from 2012 to 2015. J. Mycol. Med..

[55] Lockhart S.R., Messer S.A., Pfaller M.A., Diekema D.J. (2008). Geographic distribution and antifungal susceptibility of the newly described species Candida orthopsilosis and Candida metapsilosis in comparison to the closely related species Candida parapsilosis. J. Clin. Microbiol..

[56] Magobo R.E., Lockhart S.R., Govender N.P. (2020). Fluconazole-resistant Candida parapsilosis strains with a Y132F substitution in the ERG11 gene causing invasive infections in a neonatal unit, South Africa. Mycoses.

[57] Magobo R.E., Naicker S.D., Wadula J., Nchabeleng M., Coovadia Y., Hoosen A., Lockhart S.R., Govender N.P., van Rensburg C.J., TRAC-South Africa Group (2017). Detection of neonatal unit clusters of Candida parapsilosis fungaemia by microsatellite genotyping: Results from laboratory-based sentinel surveillance, South Africa, 2009–2010. Mycoses.

[58] Maria S., Barnwal G., Kumar A., Mohan K., Vinod V., Varghese A., Biswas R. (2018). Species distribution and antifungal susceptibility among clinical isolates of Candida parapsilosis complex from India. Rev. Iberoam. Micol..

[59] Mariangela Z., Lucieri O., Rafael M., Anna C., Andrea R. (2015). Candida parapsilosis (sensu lato) isolated from hospital located in the southeast of Brazil: Species distribution, antifungal susceptibility and virulence attributes. Int. J. Med. Microbiol..

[60] Martini C., Torelli R., De Groot T., De Carolis E., Morandotti G.A., De Angelis G., Posteraro B., Meis J.F., Sanguinetti M. (2020). Prevalence and clonal distribution of azole-resistant Candida parapsilosis isolates causing bloodstream infections in a large Italian hospital. Front. Cell. Infect. Microbiol..

[61] Mashaly G. (2014). Candida Parapsilosis Complex Species and Antifungal Susceptibility Profile in Patients of Intensive Care Units of Mansoura University Hospitals. Mans. Med. J..

[62] Melo A.S., Bizerra F.C., Freymüller E., Arthington-Skaggs B.A., Colombo A.L. (2011). Biofilm production and evaluation of antifungal susceptibility amongst clinical Candida spp. isolates, including strains of the Candida parapsilosis complex. Med. Mycol..

[63] Mesini A., Mikulska M., Giacobbe D.R., Del Puente F., Gandolfo N., Codda G., Orsi A., Tassinari F., Beltramini S., Marchese A. (2020). Changing epidemiology of candidaemia: Increase in fluconazole-resistant Candida parapsilosis. Mycoses.

[64] Miranda-Zapico I., Eraso E., Carrillo-Munoz A.J., Hernández-Molina J.M. (2011). Prevalence and antifungal susceptibility patterns of new cryptic species inside the species-complexes Candida parapsilosis and Candida glabrata among blood isolates from a Spanish tertiary hospital. J. Antimicrob. Chemother..

[65] Modiri M., Hashemi S.J., Ghazvini R.D., Khodavaisy S., Ahmadi A., Ghaffari M., Rezaie S. (2019). Antifungal susceptibility pattern and biofilm-related genes expression in planktonic and biofilm cells of Candida parapsilosis species complex. Curr. Med. Mycol..

[66] Neji S., Hadrich I., Trabelsi H., Abbes S., Cheikhrouhou F., Sellami H., Makni F., Ayadi A. (2017). Virulence factors, antifungal susceptibility and molecular mechanisms of azole resistance among Candida parapsilosis complex isolates recovered from clinical specimens. J. Biomed. Sci..

[67] Pfaller M. (2008). Global Antifungal Surveillance Group: Geographic and temporal trends in isolation and antifungal susceptibility of Candida parapsilosis: A global assessment from the ARTEMIS DISK Antifungal Surveillance Program, 2001 to 2005. J. Clin Microb..

[68] Pfaller M.A., Messer S.A., Hollis R.J. (1995). Variations in DNA subtype, antifungal susceptibility, and slime production among clinical isolates of Candida parapsilosis. Diag. Microb. Infect. Dis..

[69] Pharkjaksu S., Chongtrakool P., Suwannakarn K., Ngamskulrungroj P. (2018). Species distribution, virulence factors, and antifungal susceptibility among Candida parapsilosis complex isolates from clinical specimens at Siriraj Hospital, Thailand, from 2011 to 2015. Med. Mycol..

[70] Pinhati H.M.S., Casulari L.A., Souza A.C.R., Siqueira R.A., Damasceno C.M.G., Colombo A.L. (2016). Outbreak of candidemia caused by fluconazole resistant Candida parapsilosis strains in an intensive care unit. BMC Infect. Dis..

[71] Prażyńska M., Gospodarek E. (2014). In vitro effect of amphotericin B on Candida albicans, Candida glabrata and Candida parapsilosis biofilm formation. Mycopathologia.

[72] Puig C.R.d.A., Merino M.d.S.G., Fonseca A.D.M.P., Balbín J.A. (2021). Characterization, antifungal susceptibility and virulence of Candida parapsilosis complex isolates in a tertiary hospital in Cantabria, Northern Spain. Enferm. Infect. Microb. Clín..

[73] Pulcrano G., Panellis D., De Domenico G., Rossano F., Catania M.R. (2012). Ambroxol influences voriconazole resistance of Candida parapsilosis biofilm. FEMS Yeast Res..

[74] Raghuram A., Restrepo A., Safadjou S., Cooley J., Orloff M., Hardy D., Butler S., Koval C.E. (2012). Invasive fungal infections following liver transplantation: Incidence, risk factors, survival, and impact of fluconazole-resistant Candida parapsilosis (2003–2007). Liv. Transplant..

[75] Ramos-Martínez A., Pintos-Pascual I., Guinea J., Gutiérrez-Villanueva A., Gutiérrez-Abreu E., Díaz-García J., Asensio Á., Iranzo R., Sánchez-Romero I., Muñoz-Algarra M. (2022). Impact of the COVID-19 Pandemic on the Clinical Profile of Candidemia and the Incidence of Fungemia Due to Fluconazole-Resistant Candida parapsilosis. J. Fungi.

[76] Reiss E., Lasker B.A., Iqbal N.J., James M.J., Arthington-Skaggs B.A. (2008). Molecular epidemiology of Candida parapsilosis sepsis from outbreak investigations in neonatal intensive care units. Infect. Genet Evol..

[77] Roberto A.E.M., Xavier D.E., Vidal E.E., Vidal C.F.D.L., Neves R.P., Lima-Neto R.G.D. (2020). Rapid detection of echinocandins resistance by MALDI-TOF MS in Candida parapsilosis complex. Microorganisms.

[78] Ruiz L.d.S., Khouri S., Hahn R.C., Da Silva E.G., de Oliveira V.K.P., Gandra R.F., Paula C.R. (2013). Candidemia by species of the Candida parapsilosis complex in children’s hospital: Prevalence, biofilm production and antifungal susceptibility. Mycopathologia.

[79] Růžička F., Holá V., Votava M., Tejkalová R. (2007). Importance of biofilm inCandida parapsilosis and evaluation of its susceptibility to antifungal agents by colorimetric method. Folia Microb..

[80] Sakamoto Y., Kawabe K., Suzuki T., Sano K., Ide K., Nishigaki T., Enoki Y., Taguchi K., Koike H., Kato H. (2021). Species distribution of candidemia and their susceptibility in a single japanese university hospital: Prior micafungin use affects the appearance of candida parapsilosis and elevation of micafungin mics in non-parapsilosis candida species. J. Fungi.

[81] Sarvikivi E. (2005). Lyytik inen O, Soll DR, Pujol C, Pfaller MA, ä Richardson M, et al. Emergence of fluconazole resistance in a Candida parapsilosis strain that caused infections in a neonatal intensive care unit. J. Clin. Microb..

[82] Silva A.P., Miranda I.M., Lisboa C., Pina-Vaz C., Rodrigues A.G. (2009). Prevalence, distribution, and antifungal susceptibility profiles of Candida parapsilosis, C. orthopsilosis, and C. metapsilosis in a tertiary care hospital. J. Clin. Microb..

[83] Singh A., Singh P.K., de Groot T., Kumar A., Mathur P., Tarai B., Sachdeva N., Upadhyaya G., Sarma S., Meis J.F. (2019). Emergence of clonal fluconazole-resistant Candida parapsilosis clinical isolates in a multicentre laboratory-based surveillance study in India. J. Antimicrob. Chemother..

[84] Souza A.C.R., Fuchs B.B., Pinhati H.M., Siqueira R.A., Hagen F., Meis J.F., Mylonakis E., Colombo A.L. (2015). Candida parapsilosis resistance to fluconazole: Molecular mechanisms and in vivo impact in infected Galleria mellonella larvae. Antimicrob. Agents Chemother..

[85] Tay S.T., Na S.L., Chong J. (2009). Molecular differentiation and antifungal susceptibilities of Candida parapsilosis isolated from patients with bloodstream infections. J. Med. Microb..

[86] Thomaz D., de Almeida Jr J., Lima G., Nunes M., Camargo C., Grenfell R. (2018). An azole-resistant Candida parapsilosis outbreak: Clonal persistence in the intensive care unit of a Brazilian teaching hospital. Front. Microbiol..

[87] Thomaz D.Y., de Almeida J.N., Sejas O.N., Del Negro G., Carvalho G.O., Gimenes V.M., de Souza M.E.B., Arastehfar A., Camargo C.H., Motta A.L. (2021). Environmental clonal spread of azole-resistant Candida parapsilosis with Erg11-Y132F mutation causing a large candidemia outbreak in a Brazilian Cancer Referral Center. J. Fungi.

[88] Thomaz D.Y., Del Negro G., Ribeiro L.B., da Silva M., Carvalho G.O., Camargo C.H., de Almeida J.N., Motta A.L., Siciliano R.F., Sejas O.N. (2022). A Brazilian Inter-Hospital Candidemia Outbreak Caused by Fluconazole-Resistant Candida parapsilosis in the COVID-19 Era. J. Fungi.

[89] Tosun I., Akyuz Z., Guler N.C., Gulmez D., Bayramoglu G., Kaklikkaya N., Arikan-Akdagli S., Aydin F. (2013). Distribution, virulence attributes and antifungal susceptibility patterns of Candida parapsilosis complex strains isolated from clinical samples. Med. Mycol..

[90] Treviño-Rangel R.d.J., Garza-González E., González J.G., Bocanegra-García V., Llaca J.M., González G.M. (2012). Molecular characterization and antifungal susceptibility of the Candida parapsilosis species complex of clinical isolates from Monterrey, Mexico. Med. Mycol..

[91] Vigezzi C., Icely P.A., Dudiuk C., Rodríguez E., Miró M.S., Castillo G.D.V., Azcurra A.I., Abiega C., Caeiro J.P., Riera F.O. (2019). Frequency, virulence factors and antifungal susceptibility of Candida parapsilosis species complex isolated from patients with candidemia in the central region of Argentina. J. Mycol. Med..

[92] Wu Y., Wei D., Gong X., Shen Y., Zhu Y., Wang J., Gao Z. (2020). Initial use of voriconazole positively affects outcome of Candida parapsilosis bloodstream infection: A retrospective analysis. Transl. Pediatr..

[93] Xiao M., Fan X., Chen S., Wang H., Sun Z., Liao K., Chen S., Yan Y., Kang M. (2015). Hu 190 ZD, Chu YZ, Hu TS, Ni YX, Zou GL, Kong F, Xu YC. 2015. Antifungal 191 susceptibilities of Candida glabrata species complex, Candida krusei, Candida 192 parapsilosis species complex and Candida tropicalis causing invasive candidiasis in 193 China: 3 year national surveillance. J. Antimicrob. Chemother..

[94] Yamin D., Husin A., Harun A. (2020). Distribution of candidemia in malaysian tertiary care hospital revealed predominance of candida parapsilosis. Trop. Biomed..

[95] Zhang L., Yu S.-Y., Chen S.C.-A., Xiao M., Kong F., Wang H., Ning Y.-T., Lu M.-Y., Sun T.-S., Hou X. (2020). Molecular characterization of Candida parapsilosis by microsatellite typing and emergence of clonal antifungal drug resistant strains in a multicenter surveillance in China. Front. Microb..

[96] Gauna T.T., Oshiro E., Luzio Y.C., Paniago A.M.M., Pontes E.R.J.C., Chang M.R. (2013). Bloodstream infection in patients with end-stage renal disease in a teaching hospital in central-western Brazil. Rev. Soc. Bras. Med. Trop..

[97] Girmenia C., Martino P., De Bernardis F., Gentile G., Boccanera M., Monaco M., Antonucci G., Cassone A. (1996). Rising incidence of Candida parapsilosis fungemia in patients with hematologic malignancies: Clinical aspects, predisposing factors, and differential pathogenicity of the causative strains. Clin. Infect. Dis..

[98] Huang Y.-C., Lin T.-Y., Leu H.-S., Peng H.-L., Wu J.-H., Chang H.-Y. (1999). Outbreak ofCandida parapsilosis fungemia in neonatal intensive care units: Clinical implications and genotyping analysis. Infection.

[99] Cisneros Herreros J., Cordero Matía E. (2006). Therapeutic armamentarium against systemic fungal infections. Clin. Microb. Infect..

[100] Kohno S., Izumikawa K., Yoshida M., Takesue Y., Oka S., Kamei K., Miyazaki Y., Yoshinari T., Kartsonis N.A., Niki Y. (2013). A double-blind comparative study of the safety and efficacy of caspofungin versus micafungin in the treatment of candidiasis and aspergillosis. Eur. J. Clin. Microb. Infect. Dis..

[101] Kawai A., Yamagishi Y., Mikamo H. (2015). In vitro efficacy of liposomal amphotericin B, micafungin and fluconazole against non-albicans Candida species biofilms. J. Infect. Chemother..

[102] Wang F., Wang L. (2012). Analysis of the utilization of antifungal agents in our hospital during 2007–2009. Chin. Gen. Pract..

[103] Qin J., Yang H., Shan Z., Jiang L., Zhang Q. (2021). Clinical efficacy and safety of antifungal drugs for the treatment of Candida parapsilosis infections: A systematic review and network meta-analysis. J. Med. Microb..

[104] Zeng r., Li M., Chen Q., Wang L., Lv G., Shen Y., Cai Q., Li C., Tang R., Liu W. (2011). Dynamic study on the susceptibilities of caspofungin and micafungin to Candida species in vitro. Chin. J. Mycol..

[105] Nawaz A., Pärnänen P., Kari K., Meurman J.H. (2015). Proteolytic activity and cytokine up-regulation by non-albicans Candida albicans. Arch. Microb..

[106] Verma R., Pradhan D., Hasan Z., Singh H., Jain A.K., Khan L.A. (2021). A systematic review on distribution and antifungal resistance pattern of Candida species in the Indian population. Med. Mycol..

