# Supplementary material for: Global Prevalence of Antifungal-Resistant Candida parapsilosis: A Systematic Review and Meta-Analysis
Source: Trop Med Infect Dis. 2022 Aug 16;7(8):188. doi: 10.3390/tropicalmed7080188 (PMC9416642; doi:10.3390/tropicalmed7080188)
Supplement: Supplementary file 1 [file tropicalmed-07-00188-s001.zip › tropicalmed-1855166-supplementary.pdf]

Supplementary Materials of Global Prevalence of Antifungal Resistant Candida parapsilosis: A Systematic Review and Meta-Analysis

Dina Yamin, Mutiat Hammed Akanmu, Abbas Al Mutair, Saad Alhumaid and Ali A. Rabaan and Khalid Hajissa

Table S1. PRISMA 2020 Checklist.

Section and Topic	Item #	Checklist item	Location where item is reported
		**Title**	
Title	1	Identify the report as a systematic review.	1
		**Abstract**	
Abstract	2	See the PRISMA 2020 for Abstracts checklist.	1
		**Introduction**	
Rationale	3	Describe the rationale for the review in the context of existing knowledge.	1
Objectives	4	Provide an explicit statement of the objective(s) or question(s) the review addresses.	1
		**Methods**	
Eligibility criteria	5	Specify the inclusion and exclusion criteria for the review and how studies were grouped for the syntheses.	NR
Information sources	6	Specify all databases, registers, websites, organisations, reference lists and other sources searched or consulted to identify studies. Specify the date when each source was last searched or consulted.	2
Search strategy	7	Present the full search strategies for all databases, registers and websites, including any filters and limits used.	Table S2
Selection process	8	Specify the methods used to decide whether a study met the inclusion criteria of the review, including how many reviewers screened each record and each report retrieved, whether they worked independently, and if applicable, details of automation tools used in the process.	2
Data collection process	9	Specify the methods used to collect data from reports, including how many reviewers collected data from each report, whether they worked independently, any processes for obtaining or confirming data from study investigators, and if applicable, details of automation tools used in the process.	2-3
Data items	10a	List and define all outcomes for which data were sought. Specify whether all results that were compatible with each outcome domain in each study were sought (e.g. for all measures, time points, analyses), and if not, the methods used to decide which results to collect.	2-3
	10b	List and define all other variables for which data were sought (e.g. participant and intervention characteristics, funding sources). Describe any assumptions made about any missing or unclear information.	2-3

Section and Topic	Item #	Checklist item	Location where item is reported
Study risk of bias assessment	11	Specify the methods used to assess risk of bias in the included studies, including details of the tool(s) used, how many reviewers assessed each study and whether they worked independently, and if applicable, details of automation tools used in the process.	3
Effect measures	12	Specify for each outcome the effect measure(s) (e.g. risk ratio, mean difference) used in the synthesis or presentation of results.	3
Synthesis methods	13a	Describe the processes used to decide which studies were eligible for each synthesis (e.g. tabulating the study intervention characteristics and comparing against the planned groups for each synthesis (item #5)).	3
	13b	Describe any methods required to prepare the data for presentation or synthesis, such as handling of missing summary statistics, or data conversions.	3
	13c	Describe any methods used to tabulate or visually display results of individual studies and syntheses.	3
	13d	Describe any methods used to synthesize results and provide a rationale for the choice(s). If meta-analysis was performed, describe the model(s), method(s) to identify the presence and extent of statistical heterogeneity, and software package(s) used.	3
	13e	Describe any methods used to explore possible causes of heterogeneity among study results (e.g. subgroup analysis, meta-regression).	3
	13f	Describe any sensitivity analyses conducted to assess robustness of the synthesized results.	NA
Reporting bias assessment	14	Describe any methods used to assess risk of bias due to missing results in a synthesis (arising from reporting biases).	3
Certainty assessment	15	Describe any methods used to assess certainty (or confidence) in the body of evidence for an outcome.	NA
		**Results**	
Study selection	16a	Describe the results of the search and selection process, from the number of records identified in the search to the number of studies included in the review, ideally using a flow diagram.	3
	16b	Cite studies that might appear to meet the inclusion criteria, but which were excluded, and explain why they were excluded.	NA
Study characteristics	17	Cite each included study and present its characteristics.	5-10, Table 1
Risk of bias in studies	18	Present assessments of risk of bias for each included study.	Table S3
Results of individual studies	19	For all outcomes, present, for each study: (a) summary statistics for each group (where appropriate) and (b) an effect estimate and its precision (e.g. confidence/credible interval), ideally using structured tables or plots.	5-10
Results of syntheses	20a	For each synthesis, briefly summarise the characteristics and risk of bias among contributing studies.	NA

Section and Topic	Item #	Checklist item	Location where item is reported
	20b	Present results of all statistical syntheses conducted. If meta-analysis was done, present for each the summary estimate and its precision (e.g. confidence/credible interval) and measures of statistical heterogeneity. If comparing groups, describe the direction of the effect.	13-15
	20c	Present results of all investigations of possible causes of heterogeneity among study results.	NA
	20d	Present results of all sensitivity analyses conducted to assess the robustness of the synthesized results.	NA
Reporting biases	21	Present assessments of risk of bias due to missing results (arising from reporting biases) for each synthesis assessed.	NA
Certainty of evidence	22	Present assessments of certainty (or confidence) in the body of evidence for each outcome assessed.	NA
**DISCUSSION**			
Discussion	23a	Provide a general interpretation of the results in the context of other evidence.	18-20
	23b	Discuss any limitations of the evidence included in the review.	20
	23c	Discuss any limitations of the review processes used.	20
	23d	Discuss implications of the results for practice, policy, and future research.	20
**OTHER INFORMATION**			
Registration and protocol	24a	Provide registration information for the review, including register name and registration number, or state that the review was not registered.	NA
	24b	Indicate where the review protocol can be accessed, or state that a protocol was not prepared.	NA
	24c	Describe and explain any amendments to information provided at registration or in the protocol.	NA
Support	25	Describe sources of financial or non-financial support for the review, and the role of the funders or sponsors in the review.	NA
Competing interests	26	Declare any competing interests of review authors.	21
Availability of data, code and other materials	27	Report which of the following are publicly available and where they can be found: template data collection forms; data extracted from included studies; data used for all analyses; analytic code; any other materials used in the review.	21

From [1]. For more information, visit: http://www.prisma-statement.org/.

Table S2. Detailed Search Strategy.

Databases	Search Strategy
**PubMed**	(((((((((((((((((((((((((Antifungal[Title]) OR (Antifungals[Title])) OR ("Antifungal drug"[Title])) OR ("Antifungal susceptibility testing"[Title])) OR (Azole[Title])) OR (Fluconazole[Title])) OR (Itraconazole[Title])) OR (Itraconazole[Title])) OR (Posaconazole[Title])) OR (Voriconazole[Title])) OR ("Amphotericin B"[Title])) OR (Echinocandins[Title])) OR (Caspofungin[Title])) OR (resistance[Title]))

	OR (resistant[Title])) OR ("Drug-resistant"[Title])) OR ("drug resistance"[Title])) OR (Prevalence[Title])) OR (Prevalent[Title])) OR (Epidemiology[Title])) OR (Burden[Title])) OR (Survey[Title])) OR (Distribution[Title])) OR (Proportion[Title])) OR (surveillance[Title])) AND (((((CP[Title]) OR ("Candida parapsilosis"[Title])) OR ("C. parapsilosis"[Title])) OR (parapsilosis[Title])) OR ("parapsilosis group"[Title]))
**Scopus**	TITLE(CP OR "Candida parapsilosis" OR "C. parapsilosis" OR parapsilosis OR "parapsilosis group") AND TITLE(Antifungal OR Antifungals OR "Antifungal drug" OR "Antifungal susceptibility testing" OR Azole OR Fluconazole OR Itraconazole OR ketoconazole OR Posaconazole OR Voriconazole OR "Amphotericin B" OR Echinocandins OR Caspofungin OR resistance OR resistant OR "Drug-resistant" OR "drug resistance" OR Prevalence OR Prevalent OR Epidemiology OR Burden OR Survey OR Distribution OR Proportion OR surveillance)
**Sciencedriect**	("Candida parapsilosis" OR "C. parapsilosis" OR "parapsilosis group") AND (Antifungal OR Antifungals OR "Antifungal drug" OR "Antifungal susceptibility testing)<br><br>("Candida parapsilosis" OR "C. parapsilosis" OR "parapsilosis group") AND (resistant OR resistance OR "Drug-resistant" OR "drug resistance")<br><br>("Candida parapsilosis" OR "C. parapsilosis" OR "parapsilosis group") AND (Voriconazole OR "Amphotericin B" OR Echinocandins OR Caspofungin)<br><br>("Candida parapsilosis" OR "C. parapsilosis" OR "parapsilosis group") AND (Azole OR Fluconazole OR Itraconazole OR ketoconazole OR Posaconazole)<br><br>("Candida parapsilosis" OR "C. parapsilosis" OR "parapsilosis group") AND (Prevalence OR Prevalent OR Epidemiology OR Distribution OR Proportion)<br><br>("Candida parapsilosis" OR "C. parapsilosis" OR "parapsilosis group") AND (Burden OR Survey OR surveillance)
**Google Scholar**	allintitle: ("Candida parapsilosis" OR "C. parapsilosis" OR parapsilosis OR "parapsilosis group") (Antifungal OR Antifungals OR "Antifungal drug" OR "Antifungal susceptibility testing" OR resistant OR resistance OR "Drug-resistant" OR "drug resistance")<br><br>allintitle: ("Candida parapsilosis" OR "C. parapsilosis" OR parapsilosis OR "parapsilosis group") (Azole OR Fluconazole OR Itraconazole OR ketoconazole OR Posaconazole OR Voriconazole OR "Amphotericin B" OR Echinocandins OR Caspofungin)

allintitle: ("Candida parapsilosis" OR "C. parapsilosis" OR parapsilosis OR "parapsilosis group") (Prevalence OR Prevalent OR Epidemiology OR Burden OR Survey OR Distribution OR Proportion OR surveillance)

Table S3. Pooled *C. parapsilosis* antifungal resistance in different countries.

Subgroups		Prevalence of Antifungal resistance [95% CIs] (%)	Number of Studies Analysed	Total Number of Subjects	Heterogeneity		Publication Bias, Egger's test (*p*-value)
					I^2	*p*-value	
**Fluconazole**							
**Total**		14.4 [8.6; 20.1]	67	8373	97%	<0.0001	<0.0001
**Country**	**Spain**	18.9 [0.0–43.6]	7	890	99%	<0.01	NA
	**Italy**	12.1 [0.0–24.4]	5	705	97%	<0.01	NA
	**France**	9.2 [6.1–13.2]	1	283	NA	NA	NA
	**Finland**	0.0 [0.0–13.2]	1	26	NA	NA	NA
	**Portugal**	0.6 [0.0–3.4]	1	160	NA	NA	Na
	**Brazil**	25.3 [6.8–43.8]	15	917	98%	<0.01	0.0439
	**Argentina**	0.0 [0.0–2.3]	2	69	0%	1	NA
	**Mexico**	27.0 [0.0–73.7]	2	373	96%	<0.01	NA
	**USA**	2.3 [0.0–5.7]	4	816	85%	<0.01	NA
	**Turkey**	11.3 [2.5–20.1]	5	639	93%	<0.01	NA
	**Iran**	2.0 [0.4–3.6]	4	336	0%	0.47	NA
	**Kuwait**	2.8 [1.3–4.3]	2	556	16%	0.27	NA
	**China**	1.7 [0.0–3.7]	5	859	66%	0.02	NA
	**Japan**	0.6 [0.0–2.9]	2	90	0%	0.37	NA
	**India**	17.7 [0.0–37.1]	2	276	95%	<0.01	NA
	**Thailand**	6.2 [2.3–13.1]	1	96	NA	NA	NA
	**Malaysia**	7.5 [0.0–22.4]	2	385	97%	<0.01	NA
	**South Africa**	51.5 [20.2–82.7]	3	747	99%	<0.01	NA
	**Tunisia**	3.2 [0.0–7.4]	2	82	0%	0.32	NA
	**Egypt**	7.4 [2.4–16.3]	1	68	NA	NA	
**Amphotericin B**							
**Total**		0.1 [0.0–0.2]	61	6689	0	0.99	0.0109

**Country**	**Spain**	0.2 [0.0–0.6]	6	701	0	1	
	**Italy**	0.0 [0.0–0.5]	5	542	0	0.91	
	**France**	0.0 [0.0–1.3]	1	283	NA	NA	
	Poland	0.0 [0.0–12.3]	1	28	NA	NA	
	**Portugal**	1.2 [0.2–4.4]	1	160	NA	NA	
	**Czechia**	0.0 [0.0–17.6]	1	19	NA	NA	
	**Brazil**	0.2 [0.0–0.7]	14	852	0	0.81	0.0341
	**Argentina**	0.0 [0.0–2.3]	2	69	0	1	
	**USA**	0.0 [0.0–2.0]	2	94	0	1	
	**Turkey**	0.0 [0.0–0.5]	4	446	0	0.79	
	**Iran**	0.3 [0.0–1.1]	4	336	0	0.63	
	**Kuwait**	0.0 [0.0–0.3]	2	556	0	1	
	**China**	0.0 [0.0–0.3]	5	859	0	1	
	**Japan**	0.0 [0.0–2.1]	2	90	0	1	
	**India**	0.0 [0.0–0.6]	2	276	0	1	
	**Thailand**	0.0 [0.0–3.8]	1	96	NA	NA	
	**Malaysia**	2.9 [0.0–8.3]	2	385	86%	<0.01	
	**South Africa**	0.2 [0.0–0.5]	3	747	0	0.092	
	**Tunisia**	0.0 [0.0–2]	2	82	0	1	
	**Egypt**	0.0 [0.0–5.3]	1	68	NA	NA	
			**Voriconazole**				
**Total**		4.9 [2.3–7.6]	56	6766	91%	<0.01	0.0002
**Country**	**Spain**	7.5 [0.0–17.4]	7	890	90%	<0.01	NA
	**Italy**	5.8 [0.0–12.5]	5	690	92%	<0.01	NA
	**France**	5.3[3.0–8.6]	1	283	NA	NA	NA
	**Portugal**	0.6 [0.0–3.4]	1	160	NA	NA	NA
	**Czechia**	0.0 [0.0-17.6]	1	19	NA	NA	NA
	**Brazil**	11.7 [0.0–25.5]	10	646	96%	<0.01	NA
	**Argentina**	0.0 [0.0–2.3]	2	69	0%	1	NA
	**USA**	0.0 [0.0–10.3]	1	34	NA	NA	NA
	**Mexico**	17.2 [5.8–35.8]	1	29	NA	NA	NA
	**Turkey**	4.5 [1.6–7.3]	5	639	61%	0.04	NA
	**Iran**	0.0 [0.0–1.0]	3	216	0%	1	NA
	**Kuwait**	0.8 [0.0–2.4]	2	556	71%	0.06	NA

**China**	0.9 [0.0–2.8]	5	859	74%	<0.01	NA
**Japan**	0.0 [0.0–2.1]	2	90	0%	1	NA
**India**	0.0 [0.0–0.6]	2	276	0%	1	NA
**Thailand**	3.1 [0.6–8.9]	1	96	NA	NA	NA
**Malaysia**	1 [0.0–2.1]	2	385	0	0.5	NA
**South Africa**	19.7 [13.5–25.8]	3	747	72	0.3	NA
**Tunisia**	1.3 [0.0–4.1]	2	82	0	0.71	NA

CIs: Confidence intervals; NA: Not applicable.

Table S4. Quality assessment of the included cross-sectional studies.

**No.**	**Study ID**	**Questions Assessing Included Cross-Sectional Studies**								**Yes (%)**
		**1**	**2**	**3**	**4**	**5**	**6**	**7**	**8**	
1	Ahmadi 2020	N	N	Y	Y	Y	Y	Y	Y	75
2	Alcoceba 2022	N	Y	Y	Y	Y	Y	Y	Y	87.5
3	Alencar 2017	Y	N	Y	Y	Y	Y	Y	Y	87.5
4	Almirante 2006	Y	Y	Y	Y	N	N	Y	Y	75
5	Arastehfar 2020a	Y	Y	Y	Y	Y	Y	Y	Y	100
6	Arastehfar 2021	Y	N	Y	Y	Y	Y	Y	Y	87.5
7	Arastehfar 2020b	Y	Y	Y	Y	Y	Y	Y	Y	100
8	Asadzadeh 2017	Y	N	Y	Y	Y	Y	Y	Y	87.5
9	Asadzadeh 2008	Y	N	Y	Y	Y	Y	Y	Y	87.5
10	Ataídes 2015	Y	N	Y	Y	Y	Y	Y	Y	87.5
11	Barchiesi 2001	Y	N	Y	Y	Y	Y	Y	Y	87.5
12	Bonfietti 2012	Y	N	Y	Y	Y	Y	Y	Y	87.5
13	Cantón 2011	Y	Y	Y	Y	Y	Y	Y	Y	100
14	Castanheira 2020	Y	N	Y	Y	Y	Y	Y	Y	87.5
15	Cattana 2017	Y	N	Y	Y	Y	Y	Y	Y	87.5
16	Corzo-Leon 2021	Y	Y	Y	Y	Y	Y	Y	Y	100
17	Da Silva 2015	Y	Y	Y	Y	Y	Y	Y	Y	100
18	Davari 2020	Y	N	Y	Y	N	N	Y	Y	62.5
19	de Aguiar Cordeiro 2014	N	N	Y	Y	Y	Y	Y	Y	75
20	de Paula Menezes 2020	Y	N	Y	Y	Y	Y	Y	Y	87.5
21	Demirci-Duarte 2021	Y	N	Y	Y	Y	Y	Y	Y	87.5
22	Dizbay 2010	Y	Y	Y	Y	N	N	Y	Y	75

23	Ensieh 2017	Y	N	Y	Y	N	N	Y	Y	62.5
24	Fekkar 2021	Y	N	Y	Y	Y	Y	Y	Y	87.5
25	Fernández-Ruiz 2014	Y	Y	Y	Y	Y	Y	Y	Y	100
26	Figueiredo-Carvalho 2014	Y	N	Y	Y	Y	Y	Y	Y	87.5
27	Garcia-Effron 2012	Y	Y	Y	Y	Y	Y	Y	Y	100
28	Ge 2012	Y	N	Y	Y	Y	Y	Y	Y	87.5
29	Ghezzi 2017	Y	Y	Y	Y	Y	Y	Y	Y	100
30	Gonçalves 2010	Y	Y	Y	Y	Y	Y	Y	Y	100
31	Govender 2016	Y	Y	Y	Y	Y	Y	Y	Y	100
32	Grossman 2015	Y	N	Y	Y	Y	Y	Y	Y	87.5
33	Hilmioğlu-Polat 2018	Y	N	Y	Y	Y	Y	Y	Y	87.5
34	Hirai 2014	Y	Y	Y	Y	N	N	Y	Y	75
35	Jalel 2015	Y	N	Y	Y	Y	Y	Y	Y	87.5
36	Khan 2011	Y	N	Y	Y	Y	Y	Y	Y	87.5
37	Khodavaisy 2020	Y	Y	Y	Y	Y	Y	Y	Y	100
38	Liu 2018	Y	Y	Y	Y	N	N	Y	Y	75
39	Lockhart 2008	Y	N	Y	Y	Y	Y	Y	Y	87.5
40	Magobo 2020	Y	N	Y	Y	Y	Y	Y	Y	87.5
41	Magobo 2017	Y	N	Y	Y	Y	Y	Y	Y	87.5
42	Maria 2018	Y	Y	Y	Y	Y	Y	Y	Y	100
43	Mariangela 2015	Y	N	Y	Y	Y	Y	Y	Y	87.5
44	Martini 2020	Y	N	Y	Y	Y	Y	Y	Y	87.5
45	Mashaly 2014	Y	Y	Y	Y	Y	Y	Y	Y	100
46	Melo 2011	N	N	Y	Y	Y	Y	Y	Y	75
47	Mesini 2020	Y	Y	Y	Y	Y	Y	Y	Y	100
48	Miranda-Zapico 2011	Y	N	Y	Y	Y	Y	Y	Y	87.5
49	Modiri 2019	N	N	Y	Y	Y	Y	Y	Y	75
50	Neji 2017	Y	N	Y	Y	Y	Y	Y	Y	87.5
51	Pfaller 2008	Y	N	Y	Y	N	N	Y	Y	62.5
52	Pfaller 1995	N	N	Y	Y	Y	Y	Y	Y	75
53	Pharkjaksu 2018	Y	N	Y	Y	Y	Y	Y	Y	87.5
54	Pinhati 2016	Y	Y	Y	Y	Y	Y	Y	Y	100
55	Prażyńska 2014	Y	N	Y	Y	N	N	Y	Y	62.5
56	Puig 2021	Y	N	Y	Y	Y	Y	Y	Y	87.5

57	Pulcrano 2012	N	N	Y	Y	Y	Y	Y	Y	75
58	Raghuram 2012	Y	N	Y	Y	N	N	Y	Y	62.5
59	Ramos-Martínez 2022	Y	Y	Y	Y	Y	Y	Y	Y	100
60	Reissa 2008	N	N	Y	Y	Y	Y	Y	Y	75
61	Roberto 2020	N	N	Y	Y	Y	Y	Y	Y	75
62	Ruiz 2013	Y	N	Y	Y	Y	Y	Y	Y	87.5
63	Růžička 2007	N	N	Y	Y	N	N	Y	Y	50
64	Sakamoto 2021	Y	Y	Y	Y	N	N	Y	Y	75
65	Sarvikivi 2005	Y	N	Y	Y	Y	Y	Y	Y	87.5
66	Silva 2009	Y	N	Y	Y	Y	Y	Y	Y	87.5
67	Singh 2019	Y	N	Y	Y	Y	Y	Y	Y	87.5
68	Souza 2015	Y	N	Y	Y	Y	Y	Y	Y	87.5
69	Tay 2009	N	N	Y	Y	Y	Y	Y	Y	75
70	Thomaz 2018	N	N	Y	Y	Y	Y	Y	Y	75
71	Thomaz 2021	N	N	Y	Y	Y	Y	Y	Y	75
72	Thomaz 2022	N	N	Y	Y	Y	Y	Y	Y	75
73	Tosun 2013	N	N	Y	Y	Y	Y	Y	Y	75
74	Treviño-Rangel 2012	N	N	Y	Y	Y	Y	Y	Y	75
75	Vigezzi 2019	N	N	Y	Y	Y	Y	Y	Y	75
76	Wu 2020	Y	Y	Y	Y	N	N	Y	Y	75
77	Xiao 2015	Y	N	Y	Y	Y	Y	Y	Y	87.5
78	Yamin 2020	Y	N	Y	Y	N	N	Y	Y	62.5
79	Zhang 2020	Y	Y	Y	Y	Y	Y	Y	Y	100

1. Were the criteria for inclusion in the sample clearly defined? 2. Were the study subjects and the setting described in detail? 3. Was the exposure measured in a valid and reliable way? 4. Were objective, standard criteria used for measurement of the condition? 5. Were confounding factors identified? 6. Were strategies to deal with confounding factors stated? 7. Were the outcomes measured in a valid and reliable way? 8. Was appropriate statistical analysis used? Y=Yes; N=No; U=Unclear.

Reference

1. Page, M.J.; Moher, D.; Bossuyt, P.M.; Boutron, I.; Hoffmann, T.C.; Mulrow, C.D.; Shamseer, L.; Tetzlaff, J.M.; Akl, E.A.; Brennan, S.E.; et al. PRISMA 2020 explanation and elaboration: Updated guidance and exemplars for reporting systematic reviews. *BMJ* **2021**, *372*, n160.
